# mTORC1 coordinates an immediate unfolded protein response-related transcriptome in activated B cells preceding antibody secretion

**DOI:** 10.1038/s41467-019-14032-1

**Published:** 2020-02-05

**Authors:** Brian T. Gaudette, Derek D. Jones, Alexandra Bortnick, Yair Argon, David Allman

**Affiliations:** 1 The Department of Pathology and Laboratory Medicine, Perelman School of Medicine at the University of Pennsylvania, Philadelphia, PA 19104 USA; 2 The Children’s Hospital of Philadelphia, Philadelphia, PA 19104 USA

**Keywords:** Gene regulation, Antibodies, Marginal zone B cells, Plasma cells

## Abstract

How activated B cells build biosynthetic pathways and organelle structures necessary for subsequent robust antibody secretion is still unclear. The dominant model holds that nascent plasma cells adapt to increased antibody synthesis by activating the unfolded protein response (UPR) under the control of the transcription factor Xbp1. Here, by analyzing gene expression in activated B cells with or without plasma cell-inductive signals, we find that follicular B cells up-regulate a wide array of UPR-affiliated genes before initiating antibody secretion; furthermore, initial transcription of these loci requires the mTORC1 kinase adaptor, Raptor, but not Xbp1. Transcriptomic analyses of resting marginal zone B cells, which generate plasma cells with exceptionally rapid kinetics, reinforce these results by revealing the basal expression of UPR-affiliated mRNA networks without detectable Xbp1 activity. We thus conclude that B cells utilize mTORC1 to prepare for subsequent plasma cell function, before the onset of antibody synthesis.

## Abstract (web summary)

Antibody production in plasma cells involves the unfold protein response (UPR), but how this is regulated is not clear. Here the authors show that mTORC1 signalling but not Xbp1-mediated transcription regulation in activated B cells is important for the induction of a UPR-related transcriptome that precedes full plasma cell functions.

## Introduction

Antibody-secreting plasma cells are essential components of humoral immunity. Plasma cells contribute to host defense by secreting some 10,000 antibodies/s^1–3^. To achieve high throughput antibody synthesis newborn plasma cells must increase the size and functional capacity of the endoplasmic reticulum (ER) and the Golgi. How activated B cells deploy and regulate the biosynthetic pathways needed to expand the ER and other organelles as they differentiate into fully functional plasma cells is largely unknown.

The unfolded protein response (or UPR) is a highly conserved signal transduction pathway that drives the expression of genes needed for vigorous protein production^4^. The dominant model explaining UPR activation states that it is triggered in response to increased protein synthesis, which activates as many as three sensors in the ER membrane. The three main UPR-inducing ER sensors are Inositol-requiring trans-membrane kinase/endonuclease 1 (IRE1α), activating transcription factor-6 (ATF6), and protein kinase R-like ER kinase (PERK)^5,6^. Among these ER sensors only IRE1α is necessary and sufficient for inducing the plasma cell UPR^7–9^. Notably, when activated IRE1α uses its unique RNase activity to selectively splice transcripts encoding the transcriptional regulator Xbp1, thus creating an active transcription factor termed Xbp1s^10^. It has also been proposed that in early plasma cells Xbp1s actively promotes IgM synthesis and secretion^11^. Thus, the IRE1α/Xbp1s pathway is considered the chief regulator of the UPR in plasma cells^12,13^.

Full plasma cell differentiation also requires a unique gene regulatory network (GRN) that includes Xbp1 and the additional transcription factors Blimp1 and IRF4^13–17^. Blimp1 is a central regulator of early plasma cell differentiation, in part due to its ability to amplify immunoglobulin (Ig) gene expression to levels that activate the IRE1α/Xbp1s pathway^18^. Other work however suggests that antibody secretion can initiate without the Blimp1/Xbp1s axis^19^. For instance, Blimp1-null and Xbp1-null B cells appear to generate small numbers of cells with low antibody secretion^20,21^, key proteins of the UPR have been detected upon plasma cell induction in a transformed B cell line^22^, and spliced Xbp1 transcripts can be detected without IgM synthesis^23^. However, the timing with which these pathways are implemented in activated B cells and pre-plasma cells, and the implications of this timing for understanding UPR inductive mechanisms, remain unclear.

The mTORC1 kinase complex may play a key and unique role in UPR activation, but the mechanisms and timing with which mTORC1 contributes to this process are uncertain. It should also be noted, while mTORC1 clearly regulates protein translation^24^, mTORC1 may also regulate mRNA abundance for certain genes via poorly understood mechanisms^25^. We recently reported that the ER chaperone and IRE1α regulator BiP/Grp78^26^ is induced very early in plasma cell differentiation in a mTORC1-dependent manner^27^. Other work suggests that deregulated mTORC1 activity promotes suboptimal antibody secretion despite loss of Xbp1^28^. Importantly, while the latter report suggests that mTORC1 can substitute to some degree for Xbp1s via a poorly understood mechanism, it does not establish a role for mTORC1 signaling in the natural implementation of the UPR or distinguish between induction of UPR-affiliated genes versus frank UPR activation.

Here, we report transcriptomic data on recently activated B cells including in cells lacking Xbp1 or mTORC1 function. Our results reveal that mTORC1 drives the expression of a large battery of UPR-affiliated mRNAs, well before the onset of plasma cell function and independently of Xbp1s. We therefore conclude that activated B cells use mTORC1 to anticipate subsequent antibody synthesis.

## Results

### B cell differentiation kinetics

**Fig. 1:**
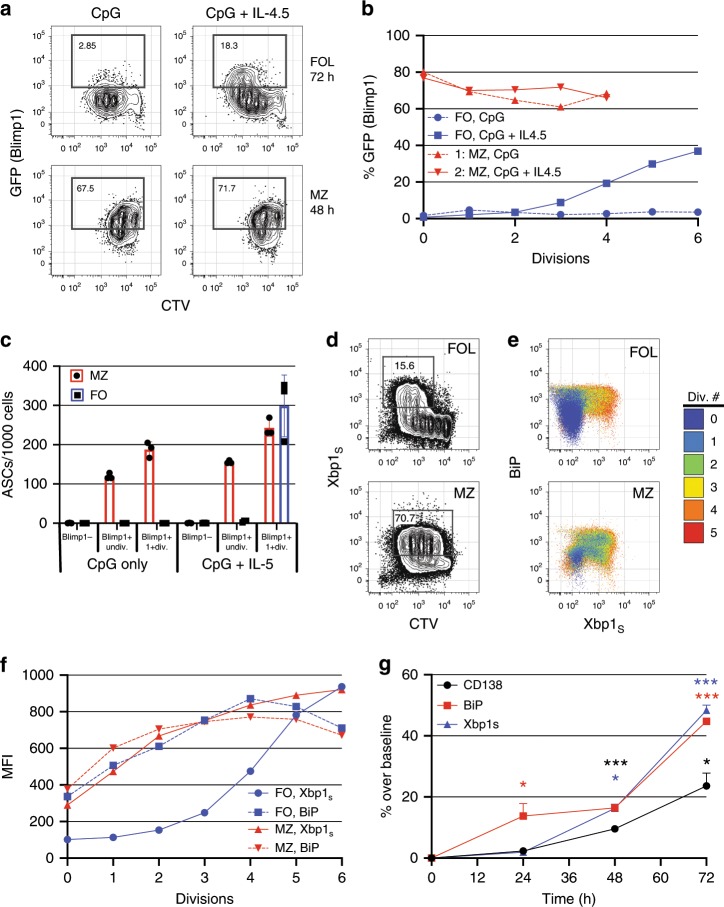
Control of B cell activation versus differentiation. **a** Celltrace violet (CTV) labeled follicular and MZ splenic B cells were purified from B6.Blimp1^+/GFP^ adults by cell sorting, stimulated for the indicated duration with CpG with and without IL-4 and IL-5, and assayed by flow cytometry for GFP expression and CTV dilution. **b** GFP expression as a function cell division for each cell and condition. **c** Follicular and MZ B cells from additional B6.Blimp1^+/GFP^ adults were stimulated as in **a**, then GFP^+^ and GFP^−^ cells that had or had not completed at least one cell division sorted into ELISPOT plates to quantify frequencies of total antibody-secreting cells (ASCs). Data is shown as individual data points, mean(bar) and SEM. **d** MZ and follicular B cells from a C57BL/6 adult were stimulated with CpG + IL-4,5 as in **a** for 72 h, then intracellular Xbp1_S_ levels evaluated as a function of CTV dilution. **e**, **f** MZ and follicular B cells were stimulated and analyzed as in **d** except for the additional use of an anti-BiP antibody. Shown are cell fractions back gated based on the indicated division number **e**, with MFIs graphed as a function of division number in **f**. **g** Follicular B cells were stimulated with CpG + IL-4/5 for the indicated times, and expression of the indicated proteins evaluated by flow cytometry and presented as percent of cells increased from baseline expression. Statistic indicates change in consecutive time points. Data is shown as mean and SEM (*p < 0.05, **p < 0.01, ***p < 0.001; repeated measures ANOVA, Tukey HSD post-test). Flow cytometry in **a**, **d** and **e** are representative experiments of at least three experimental replicates. Source data are provided as a Source Data file.

Our objective was to study biochemical events associated with plasma cell differentiation before Blimp1 induction in both follicular and marginal zone (MZ) B cells. MZ B cells generate plasma cells with accelerated kinetics compared to follicular B cells^29^, and are thought to play critical roles in humoral immunity to blood-borne microbes^30^. Hence to probe for insights into the molecular regulation of early plasma cell differentiation we compared differentiation kinetics and UPR-related gene expression in both follicular and MZ B cells. To this end, we first established a simple in vitro stimulation assay allowing direct comparisons of changes in specific transcript abundance in activated B cells versus early plasma cells. Splenic follicular B cells harvested from adult B6.Blimp1^+/GFP^ reporter mice stimulated with the TLR-9 ligand CpG proliferated robustly over 72 h, but failed to yield antibody-secreting Blimp1^+^ cells unless also stimulated with IL-4 and/or IL-5 (Fig. 1a–c, Supplementary Fig 1a). By contrast, CpG-stimulated MZ B cells yielded plasma cells within 48 h with fewer cell divisions, and without intentional cytokine addition (Fig. 1a–c). Importantly, we did not detect active antibody synthesis in freshly isolated MZ B cells (Supplementary Fig. 1b). Consistent with the idea that Xbp1 splicing occurs downstream of Blimp1 activation^18^, for follicular B cells we only detected Xbp1s after four cell divisions, quite similar to the onset of expression for Blimp1 and the canonical plasma cell marker CD138 (Syndecan-1) (Fig. 1a, d)^31^. Notably, the induction kinetics for Xbp1_S_ protein in follicular B cells were clearly delayed compared to BiP: Whereas Blimp1^+^ and Xbp1_S_^+^ cells were detected only in post-division cells and only well after 24 h, BiP^+^ cells were evident within 24 h and in undivided cells (Fig. 1e–g, Supplementary Fig 1c). These data raised the possibility that UPR-associated genes in addition to BiP are induced before full Xbp1-regulated implementation of the UPR.

### Basal UPR gene expression in MZ B cells

**Fig. 2:**
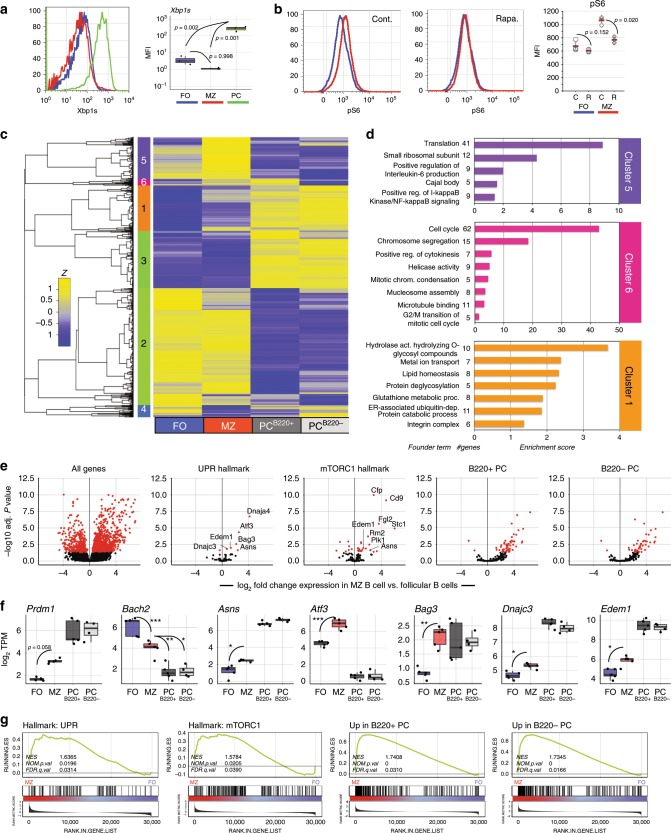
Basal UPR gene expression in resting MZ B cells. **a** C57BL/6 mouse splenocytes were stained for intracellular Xbp1s. Gating criteria are shown in Supplementary Fig. 1. Shown is a representative histogram and MFI of three animals, which are summarized as the mean (line), 75% CI (box), and 95% CI (whisker) and individual points. One-way ANOVA with Tukey post-test was performed to generate *p* values. **b** Intracellular phosphorylated S6 protein staining was performed on follicular (FO) and MZ B cells from mice treated with rapamycin or vehicle control every other day for a total of four treatments. Counts as percent of maximum are displayed with quantification on right. *p* value is two-tailed Student’s *t*-test. Summary data for all animals presented as individual data points, mean and SEM. **c**–**g** RNA-seq was performed on splenic follicular and MZ B cells and short-lived (B220^+^) and long-lived (B220^−^) BM PCs from five individual B6.Blimp^+/GFP^ adults. **c** Differentially expressed genes were averaged by group and gene co-regulation was determined by hierarchical clustering by Pearson correlation with a grouping cutoff (*k*) of six chosen using best of 26 indices by NbClust^71^. Gene expression is averaged by group (*n* = 5) for clarity and displayed as *z* score across each row. **d** Gene ontology clustering enrichment analysis of selected co-expression clusters is shown. Indicated is the founder term for each GO term cluster followed by gene numbers for that term. Bar length indicates the enrichment score for the GO cluster. **e** Volcano plots showing genes differentially expressed in MZ B cells over follicular B cells for all genes (first panel), UPR hallmark genes, mTORC1-signaling hallmark genes, the top 250 upregulated genes in B220^+^ BM PCs versus follicular B cells and the top 250 genes upregulated in B220^−^ BM PCs versus follicular B cells. Genes are color coded by adjusted *p*-value (red = *p* < 0.05). Adjusted *p*-value is BH-adjusted (eBayes method—Limma). **f** Log_2_ TPM expression magnitude of selected genes is shown as mean (line), 75%CI (box) and 95%CI (whisker) as well as individual data points (jitter). (*p < 0.05, **p < 0.01, ***p < 0.001; differential expression statistic: BH-adjusted *p*-value—eBayes method—Limma). **g** GSEA was performed using the BROAD UPR and mTORC1-signaling hallmark gene sets as well as B220^+^ and B220^−^ upregulated gene sets described in **e** comparing MZ B and follicular B cells. FDR-q values computed using 1000 geneset permutations. Source data are provided as a Source Data file.

Next, we tested for mTORC1 activation and the expression of UPR-affiliated genes in resting and activated B cells. Given the relative ease with which MZ B cells yield plasma cells (Fig. 1a–c)^29^, we first probed for evidence of constitutive Xbp1s and mTORC1 activity and expression of UPR-affiliated genes in naïve follicular and MZ B cells. Whereas we failed to detect Xbp1s protein in freshly isolated cells from either population (Fig. 2a), we did detect phosphorylation of the canonical mTORC1 substrate S6 (pS6) in MZ B cells; and pS6 levels were reduced to background following in vivo blockade of mTORC1 with rapamycin (Fig. 2b).

Next we used RNA-seq to compare global mRNA transcript abundance in freshly isolated splenic follicular and MZ B cells together with immature B220^+^ and mature B220^−^ bone marrow (BM) plasma cells, with all populations twice sorted from 4 to 5 individual mice (see Supplementary Fig. 1d, e). Although past microarray data revealed differential gene expression between follicular and MZ B cells^32^, due to its increased sensitivity^33^, we reasoned that RNA-seq analyses would reveal additional insights into mechanisms underlying the plasma cell poised state of MZ B cells, especially when compared directly to immature plasma cells. Of note, we developed an in-house RNA-seq data set for each population because this approach allowed us to track gene expression changes in many cell types and conditions across multiple biological replicates.

Our RNA-seq analyses revealed 1205 differentially regulated genes between freshly isolated follicular and MZ B cells (Supplementary Fig. 2a, b). Hierarchical clustering of the 7249 genes differentially expressed between all four cell types groups without respect to Ig transcripts resolved distinct clusters of genes for which transcript abundance was uniquely shared between MZ B cells and both plasma cell populations (Clusters #1, #6), although abundance for most of these transcripts was lower in MZ B cells compared to plasma cells (Fig. 2c). Cluster #1 was enriched for genes encoding proteins associated with protein modification pathways, lipid homeostasis, and ER-associated protein catabolism, and Cluster #6 for loci associated with mitosis (Fig. 2d). Notably, MZ B cells also expressed a unique mRNA cluster (Cluster #5) enriched for gene products involved in protein translation and ribosome synthesis (Fig. 2c, d). Direct comparison of differentially expressed genes between follicular and MZ B cells revealed that many genes belonging to the canonical UPR and mTORC1-signaling hallmark gene sets are upregulated in resting MZ B cells (Fig. 2e). We also prepared gene signatures consisting of the top 250 genes upregulated in both immature (B220^+^) and long-lived (B220^−^) BM plasma cells compared to follicular B cells and observed a large number of these genes to be upregulated in MZ B cells (Fig. 2e). Evaluation of individual transcripts confirmed that resting MZ B cells express an array of UPR-associated loci. These included genes encoding the regulatory chaperones p58^IPK^ (*Dnajc3*) and DnaJ/Hsp40 (*Dnaja4*), asparagine synthetase (*Asns*), the regulator of the endoplasmic-reticulum-associated protein degradation (ERAD) pathway Edem1 (*Edem1*)^34^, and the transcriptional regulator of the stress response ATF3 (*Atf3*) (Fig. 2e, f). We also observed modest increases in mRNA abundance for Blimp1 (*Prdm1*), and modest decreases in transcript abundance for Bach2 (*Bach2*), a negative regulator of Blimp1 transcription^35^ (Fig. 2f). Importantly, other components of the canonical plasma cell GRN were not significantly changed; for instance, we observed no differences in mRNA levels between follicular B cells and MZ B cells for *Pax5* or *Bcl6* (Supplementary Fig. 2d).

We also employed gene set enrichment analysis (GSEA) to examine changes in transcriptional pathway activation to probe for coordinated changes in functionally related genes^36^. We compared MZ B cells to follicular B cells using hallmark gene sets for the UPR and mTORC1 signaling^36,37^. These results revealed significant enrichment for canonical UPR and mTORC1-signaling targets in resting MZ B cells (Fig. 2g). We then examined expression of the plasma cell program in MZ B cells using our in-house generated immature and long-lived plasma cell signatures and observed significant skewing toward in favor of MZ B cells over follicular B cells (Fig. 2g). Leading edge analysis confirmed that several UPR targets as well as *Prdm1* are components of a core plasma cell gene expression signature in MZ B cells (Supplementary Fig. 2e). We conclude that resting MZ B cells express many genes associated with plasma cell differentiation including several canonical UPR targets expressed in mature long-lived plasma cells, despite the absence of antibody secretion and Xbp1s typical of functional plasma cells. Therefore the transcription of UPR-affiliated genes can occur without full plasma cell function.

### UPR target gene activation in pre-plasma cells

**Fig. 3:**
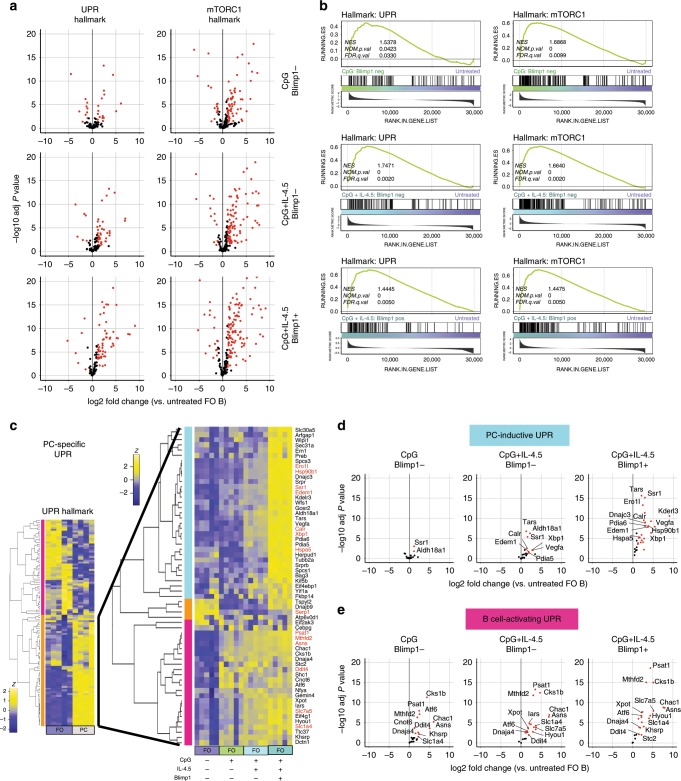
Development of a plasma cell-inductive UPR signature. CTV-labeled splenic follicular B cells were sorted from five B6.Blimp1^+/GFP^ adults, and cultured with CpG with and without IL-4 + IL-5 for 72 h. RNA-seq was performed on cDNA prepared from 4th division GFP/Blimp1^−^ cells treated with CpG or CpG plus IL-4/5, 5th division GFP/Blimp1^+^ CpG plus IL-4/5 cells, as well as freshly isolated follicular and MZ B cells (Supplementary Fig. 3). **a** Volcano plots display differential expression of UPR and mTORC1-signaling hallmark gene sets comparing the indicated treatment groups to freshly isolated follicular B cells (group *n* = 5 animals). Genes are color coded by the differential expression statistic *p* < 0.05, log_2_ fold change > 1 (BH-adjusted *p*-value—eBayes method—Limma). **b** GSEA was performed using the BROAD UPR and mTORC1-signaling hallmark gene sets comparing indicated treatment groups and freshly isolated follicular B cells. FDR-q values computed using 1000 geneset permutations (group *n* = 5 animals). **c** RNA-seq comparing UPR hallmark gene expression in BM B220^−^ plasma cells (PCs) to splenic follicular B cells was used to identify PC-specific UPR genes. The expression of these genes was then assessed across in vitro-activated and differentiated follicular B cells to determine those genes induced by B cell activation (magenta) and those requiring PC-inductive stimulus (cyan). Indeterminate genes (orange) were omitted. Those genes printed in red are contained within both the BROAD UPR and mTORC1 signaling gene sets. **d**, **e** Volcano plots display differential expression of gene sets defined in **c** for indicated treatment groups compared to freshly isolated follicular B cells (group *n* = 5 animals). Genes are color coded by the differential expression statistic *p* < 0.05, log_2_ fold change > 1 (BH-adjusted *p*-value—eBayes method—Limma).

We sought to test whether activated follicular B cells experience a UPR-enriched gene expression profile before the onset of plasma cell function similar to resting MZ B cells. In light of the observed mTORC1 activity in unstimulated MZ B cells, we first assessed the activation of mTORC1 in in vitro-activated B cells. We observed robust S6 phosphorylation in cells treated with CpG with or without cytokine (Supplementary Fig 3a). It has been shown that TLR9 and even IL-5 receptor signaling can activate mTORC1 via the PI3 kinase-AKT pathway^38–40^. We therefore evaluated the expression of phosphorylated AKT in these cells. We observed significant increases in phosphorylated AKT in cells treated with CpG with or without adding cytokines, and in cells treated with IL-5 alone (Supplementary Fig. 3b). Next, we prepared RNA-seq libraries from B6.Blimp1^+/GFP^-derived follicular B cells stimulated for 72 h with either activating (CpG alone) or plasma cell-inductive (CpG + IL-4,5) conditions. We compared activation of the UPR and mTORC1 pathways between Blimp1^−^ CpG-treated B cells, Blimp1^−^ CpG + IL-4,5-treated B cells, and Blimp1^+^ CpG + IL-4,5-treated plasma cells, and witnessed differential activation of the UPR pathway genes by both differential expression and GSEA analyses (Fig. 3a, b, Supplementary Fig. 3c). While robust activation of hallmark mTORC1-signaling genes was evident in all stimulation groups, differences in the quality and scale of the expression of UPR-affiliated genes were evident (Fig. 3a, b). To define the plasma cell-specific UPR program more directly, we first identified UPR hallmark genes up-regulated in long-lived B220^−^ BM plasma cell relative to freshly isolated follicular B cells (Fig. 3c). We then compared the expression of plasma cell-specific UPR genes across the three in vitro-induced populations above along with resting follicular B cells. Notably, this strategy revealed that 24 of these genes were induced in Blimp1/GFP^−^ B cells by activating conditions (magenta sidebar), whereas an additional 33 UPR-affiliated genes were observed in Blimp1/GFP^−^ cells experiencing plasma cell-inductive conditions (cyan sidebar) (Fig. 3c–e). As expected, expression of both gene sets was also observed in Blimp1/GFP^+^ cells (Fig. 3d, e). Given that the expression of many UPR-affiliated genes preceded XBP1s protein expression, we probed for bona fide UPR function in stimulated B cells by evaluating the activation status of several canonical ER sensors. Western blot analyses revealed no evidence for PERK, ATF6, or IRE1 activity in activated B cells, whereas, with the exception of PERK, each was clearly activated in actual plasma cells (Supplementary Fig. 3c). We did however observe upregulation of p58IPK in stimulated B cells as well as plasma cells. The latter finding is consistent with past work suggesting that p58IPK plays an important role in early plasma cell function by preventing PERK-mediated inhibition of protein translation^7^ (Supplementary Fig. 3c). Altogether these findings suggest that the transcription of numerous UPR-affiliated genes is increased in activated B cells, before initiation of UPR function in early plasma cells.

**Fig. 4:**
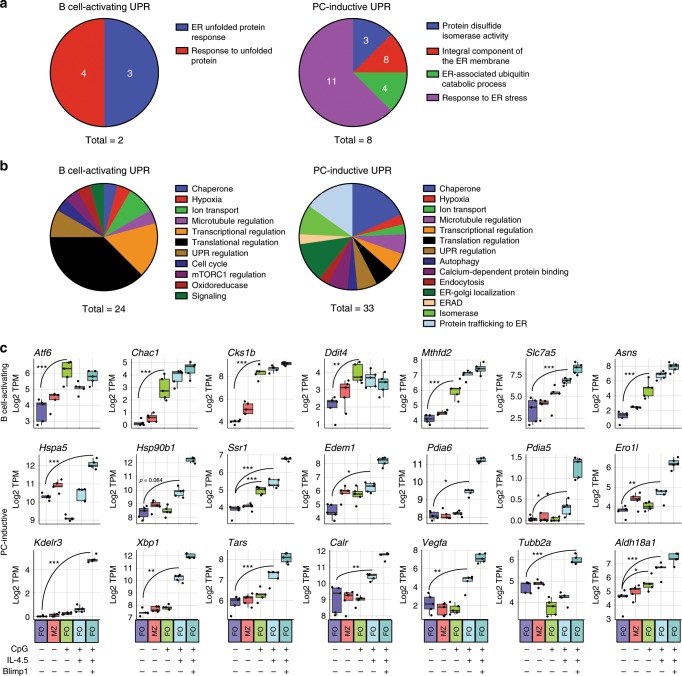
Qualitative differences in activation induced and plasma cell-differentiation induced UPR gene sets. **a** Gene sets defined in Fig. 3c were subjected to gene ontology (GO) cluster-enrichment analysis. Biological process, cellular compartment, and molecular function annotation was used to determine clusters of enriched terms. Only those terms with a *p* value < 0.05 were used in clustering. The term with maximum number of contained genes was chosen as the founder term and the number of genes is shown in the wedge. **b** Each gene in the gene sets defined in Fig. 3c was annotated by functional category. Wedge indicates the number of genes in the term as part of the total genes in the gene set. **c** From the RNA-seq dataset described in Fig. 3, the Log_2_ TPM magnitude of expression of selected genes in each gene set is shown as mean (line), 75%CI (box) and 95%CI (whisker) as well as individual data points (jitter). (*p < .05, **p < .01, ***p < .001; differential expression statistic: BH-adjusted *p*-value—eBayes method—Limma). PC plasma cell. Source data are provided as a Source Data file.

We hypothesized that B cell activation without plasma cell inductive stimuli induces the expression of UPR components that amplify translation and modify nascent proteins, whereas UPR-affiliated genes observed in Blimp1/GFP^−^ pre-plasma cells are affiliated with expansion of relevant organelles needed for eventual full plasma cell function. To test this hypothesis, we first performed gene ontology (GO) functional annotation clustering analysis. We observed that while both groups were enriched for the expected UPR and ER stress GO terms, the plasma cell-inductive group exhibited enrichment for more functionally diverse terms, such as protein disulfide isomerases, ER membrane components, and ERAD functions (Fig. 4a). Closer examination of the molecular and cellular functions of members of these two groups of genes revealed further qualitative distinctions. More than half of the B cell-activating UPR group contained genes connected to the regulation of transcription and translation or sensors of misfolded proteins. By contrast, the plasma cell-inductive UPR segment in pre-plasma cells was enriched for genes important for ER-genesis and building of the secretory apparatus, including chaperones, isomerases, and gene products that regulate protein trafficking, ER–golgi localization, and the ERAD pathway (Fig. 4b, Supplementary Table 1).

Examination of specific genes provided additional clues about how activated B cells establish biosynthetic pathways needed for robust antibody secretion. Transcripts that were readily induced by B cell activation alone included those encoding sensors of misfolded proteins, such as ATF6 (*Atf6*), ion transport regulators such as cation transport-regulator-1 (*Chac1*), regulation of cell cycle (CDC28 protein kinase 1b (*Cks1b*)), the negative regulator of mTORC1 activity known as DNA damage-inducible gene 4 (*Ddit4*), and methylenetetrahydrofolate dehydrogenase (*Mthfd2*). Furthermore, a plurality of these genes (8 of 24) have been connected to the regulation and maintenance of protein translation. These included the amino acid transporter encoded by *Slc7a5*, and asparagine synthase (*Asns*) (Fig. 4c). In contrast, among transcripts up-regulated in Blimp1/GFP^−^ pre-plasma cells, more than half encode proteins involved in protein secretion. These encompassed chaperones including BiP (*Hspa5*) and GRP94 (*Hsp90b1*), regulators of ER trafficking, such as the signal sequence receptor *Ssr1*, facilitators of disulfide bond formation including protein disulfide isomerase A6 (*Pdia6*) and the oxidoreductase *Ero1l*, ERAD regulator *Edem1*, and several regulators of ER-golgi localization such as the ER-lumen protein-retaining receptor *Kdelr3* (Fig. 4c). We also witnessed up-regulation of full-length Xbp1 transcripts in these cells (Fig. 4c). Importantly, both of these functional groups were initiated preceding activation of a Blimp1-dependent canonical plasma cell program.

### UPR-related gene expression in activated B cells in vivo

To extend our results we examined gene expression in early activated B cells and plasma cells in vivo. To this end, we immunized B6.Blimp1^+/GFP^ mice with the hapten-carrier conjugate nitrophenyl-lipopolysaccharide (NP-LPS). The kinetics of plasma cell induction in response to haptenated LPS are well-established^41^, and NP-specific B cells and plasma cells are readily purified by cell sorting using NP-conjugated fluorphores^42^. As expected, 4 days post-immunization we readily observed both NP-binding Blimp1^−^ CD138^−^-activated B cells and Blimp1^+^ CD138^+^ plasma cells (Supplementary Fig. 4a). Evaluation of BiP and XPB1s expression by flow cytometry on day 4 revealed a small increase in BiP staining in activated B cells relative to resting IgD^+^ B cells, whereas as expected we only detected XBP1s expression in bona fide plasma cells (Supplementary Fig. 4a–c).

**Fig. 5:**
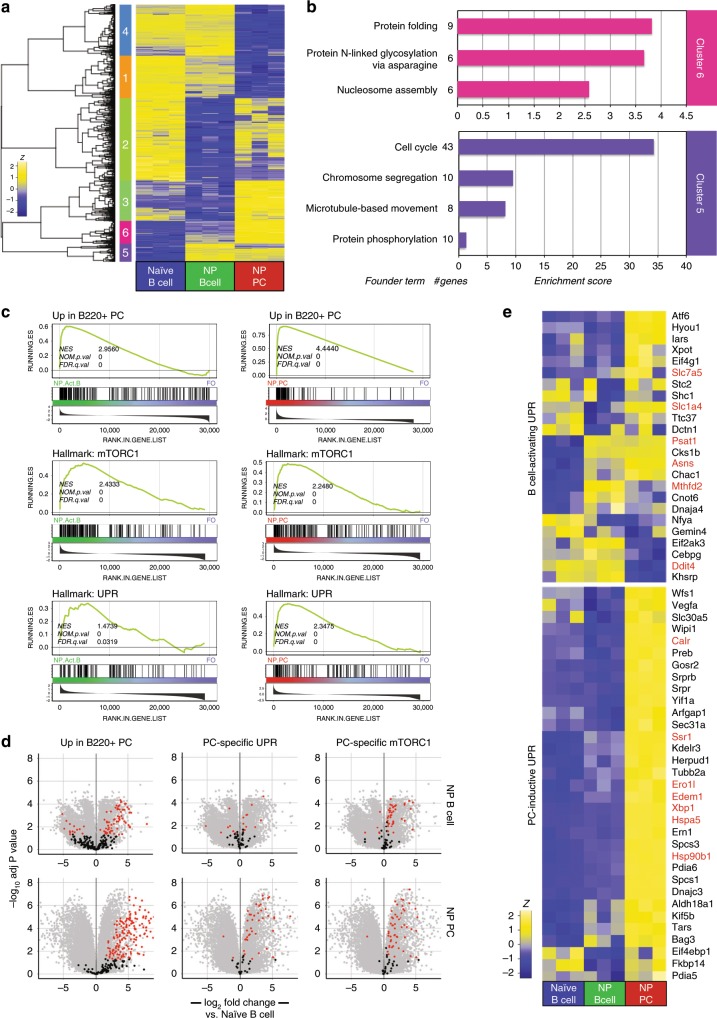
Induction of UPR-related genes in activated B cells in vivo. B6.Blimp1^+/GFP^ mice were immunized with 50 μg NP-LPS intraperitoneally. Naïve (IgD^high^, Dump[CD4, CD8α, F4/80, Ter199]^−^, B220^+^, NP^−^), antigen-specific activated B cells (IgD^−^, Dump[CD4, CD8α, F4/80, Ter199]^−^, GFP^−^, CD138^−^, B220^+^, NP^+^), and antigen-specific plasma cells (PCs) (IgD^−^, Dump[CD4, CD8α, F4/80, Ter199]^−^, GFP^+^, CD138^+^, NP^+^) were twice sorted for RNA and cDNA preparation and RNA-sequencing. **a** 1840 differentially expressed genes between the three groups were defined by BH-adjusted *p*-value < 0.01 and log_2_ fold change ≥ 3 and hierarchically clustered by Pearson correlation and broken into six clusters as determined by within sum of squares (elbow) method. **b** Gene ontology clustering enrichment analysis of selected co-expression clusters is shown. Indicated is the founder term for each GO term cluster followed by the number of genes represented in that term. Bar length is an indication of the enrichment score for the GO cluster. **c** GSEA was performed using the BROAD UPR and mTORC1-signaling hallmark gene sets as well as the B220^+^ PC gene signature defined in Fig. 2e comparing indicated groups. FDR-q values computed using 1000 geneset permutations (*n* = 3 animals). **d** Volcano plots display the differential gene expression between NP-specific B cells and PCs versus IgD^+^ B cells. Genes in red indicate adjusted *p* < 0.05 and Log_2_ fold change at least 0.5. Indicated genesets are overlayed on all genes in gray. **e** Gene expression for the PC-sp**e**cific UPR genesets defined in Fig. 3 are shown. Indicated in red are genes found in both the mTORC1 signaling and UPR hallmark genesets.

To examine gene expression, NP-binding cells within both populations were twice-sorted from three individuals and subjected together with naïve IgD^+^ B cells to RNAseq. Analysis of 1840 genes that were differentially expressed (>3-fold) in activated NP-specific B cells reveal significant enrichment for genes that encode proteins that regulate protein folding and glycosylation (Fig. 5a, b, Supplementary Fig. 4d). Furthermore, we observed strong enrichment for both the mTORC1 signaling and UPR hallmark genesets in NP-specific B cells (Fig. 5c). Specifically, several members of the plasma cell-specific UPR and mTORC1 genesets including the isomerase encoded by Ero1l, the signal sequence receptor Ssr1, and ERAD component Edem1, although generally and as expected mRNA abundance for these genes was lower than that observed for Blimp1^+^ CD138^+^ cells (Fig. 5d, e). We conclude that numerous canonical mTORC1 and UPR-affiliated genes are expressed in activated antigen-specific B cells preceding plasma cell differentiation in vivo.

### Impact of Xbp1 mutation

**Fig. 6:**
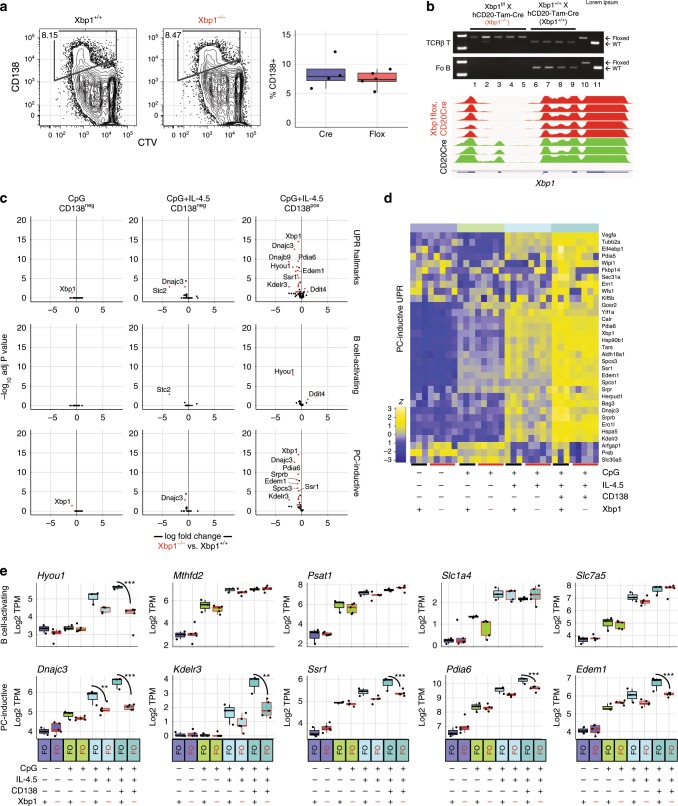
Xbp1-independent early activation of UPR gene expression. Follicular B cells were prepared from either hCD20-TAM-Cre (*n* = 3) or Xbp1^fl/fl^;hCD20-TAM-Cre (*n* = 5) animals after 10 days of tamoxifen diet. Sorting was performed as in Supplementary Fig. 1e. These cells were then in vitro activated and differentiated as in Fig. 3 using CD138 staining to identify bona fide plasma cells (PCs). **a** CTV-labeled follicular B cells from the mice described in **a** were stimulated with CpG + IL-4/5 for 72 h, stained for CD138 expression, and analyzed. All plots show viable cells only. (**b**, upper panel) CD20-TAM-Cre;Xbp1^fl/fl^ (1–5) and CD20-TAM-Cre;Xbp1^+/+^ (6–9) female adults from above. Genomic DNA from purified follicular B cells (CD19^+^, AA4.1^−^, CD23^+^, CD21/35^int^) and CD19^−^, TCRβ^+^ T cells was used to detect WT and floxed Xbp1 by PCR. Genotypes shown at right. Note loss of PCR product in CD19^+^ CD20-TAM-Cre;Xbp1^fl/fl^ cells. Lanes 10 and 11 are PCR products from control tail DNA from Xbp1^f^ and C57BL6 mice, respectively. (**b**, lower panel) Alignment tracks of RNA-seq reads at the Xbp1 locus from Blimp^−^ CpG + IL-4,5-stimulated follicular B cells from the indicated individual mice displaying the selective loss of the floxed exon 2. **c** Volcano plots display differential expression of UPR hallmark genes and the gene sets defined in **c** for indicated treatment groups compared to freshly isolated follicular B cells (group *n* = 3–5 animals). Genes are color coded by the differential expression statistic *p* < 0.05, log_2_ fold change > 1 (BH-adjusted *p*-value—eBayes method—Limma). **d** Expression of PC-inductive UPR genes as defined in Fig. 3c are shown for each animal and in vitro-treatment group as the *z* score across each row. **e** Log_2_ TPM magnitude of expression of selected genes in each gene set is shown as mean (line), 75%CI (box) and 95%CI (whisker) as well as individual data points (jitter) (*p < 0.05, **p < 0.01, ***p < 0.001; differential expression statistic: BH-adjusted *P*-value—eBayes method—Limma). Source data are provided as a Source Data file.

To determine if pre-differentiation priming of the ER is indeed Xbp1-independent, we bred mice harboring a Xbp1 allele wherein exon 2 is flanked by loxP sites (Xbp1^f/+^) with mice possessing a CD20-regulated tamoxifen-induced CD20-Cre fusion protein (hCD20-Tam-Cre). Of note, it has been reported that Xbp1-deficient B cells can be induced to secrete antibody at readily detectable albeit suboptimal quantities^21^, but the impact of Xbp1 loss-of-function on early induction of a broad coalition of UPR components has not been reported. We fed adult hCD20-Tam-Cre; Xbp1^f/f^ and hCD20-Tam-Cre;Xbp1^+/+^ mice tamoxifen for 10 days, and then prepared, stimulated, and analyzed splenic follicular B cells from individual mice. In vitro stimulation of cells from 3 to 5 individuals of each genotype showed no difference in their ability to generate CD138^+^ cells after stimulation for 72 h with CpG + IL-4,5 (Fig. 6a). The upregulation of CD138 was consistent both kinetically and by cell division number with the upregulation of Blimp1 and Xbp1s expression (see Fig. 1a, d). Importantly, PCR analysis of genomic DNA prepared from unstimulated B and T cells and parallel RNA-seq analyses of stimulated B cells from each individual both revealed selective loss of exon 2 in B cells from hCD20-Tam-Cre; Xbp1^f/f^ mice (Fig. 6b). We next performed RNA-seq with Xbp1 sufficient and deficient follicular CD138^−^ B cells induced via activating (CpG alone) or plasma cell inductive (CpG + IL4,5) conditions. We also analyzed CD138^+^ plasma cells from the latter group. These data indicated that both the B cell activating and plasma cell-inductive UPR program were activated robustly despite loss of Xbp1 (Supplementary Fig. 5). Indeed, among activated CD138^−^ cells with minor exceptions there was no difference in the up-regulation of UPR genes in any stimulation group (Fig. 6c, d). The only gene differentially expressed gene between the CpG-treated groups was Xbp1 itself, and only the Hsp40 member chaperone and PERK regulator *Dnajc3* and stanniocalcin 2 (*Stc2*) were differentially expressed in CD138^−^ cells with CpG + IL-4,5 stimulation (Fig. 6c, d). Further and in contrast, among induced CD138^+^ cells we observed a unique and partial dependence on Xbp1 for maximal upregulation of the UPR (Fig. 6c, d). Whereas only the hypoxia-regulated gene *Hyou1* was dependent on Xbp1 for maximal expression after expression of CD138 in the B cell-activating UPR group, as many as 15 of the genes in the plasma cell inductive UPR group were further enhanced by Xbp1 activity following CD138 expression. These broadly included chaperones (*Dnajc3*), protein-trafficking effectors (*Kdelr3, Ssr1*), isomerases (*Pdia6*), and the ERAD modulator *Edem1* (Fig. 6e). These data indicate that both regulation of basic cellular processes of transcription and translation and adaptive ER organelle genesis occur in an Xbp1-independent manner and only the latter is then amplified and maintained by Xbp1 following differentiation.

### mTORC1-dependent deployment of UPR gene expression

We next evaluated the impact of inducing B cell-specific mutation of the essential mTORC1 adaptor Raptor on early induction of UPR gene expression. To this end we prepared B cells from individual Raptor^f/f^;hCD20-Tam-Cre and control Raptor^+/+^;hCD20-Tam-Cre adults (three mice per genotype per time point) that had been exposed to tamoxifen for 10 days as described above (Supplementary Fig. 6a). Splenic follicular B cells from each mouse were stimulated for 24, 48, or 72 h using plasma cell-inductive (CpG + IL-4,5) conditions, and RNA-seq was performed on sorted viable cells from each sample. Notably, while we did not observe induction of CD138 by Raptor-deficient B cells, these cells did exhibit increased viability in vitro, indicating that loss of Raptor did not lead to increased cell death in vitro (Supplementary Fig. 6a–c).

**Fig. 7:**
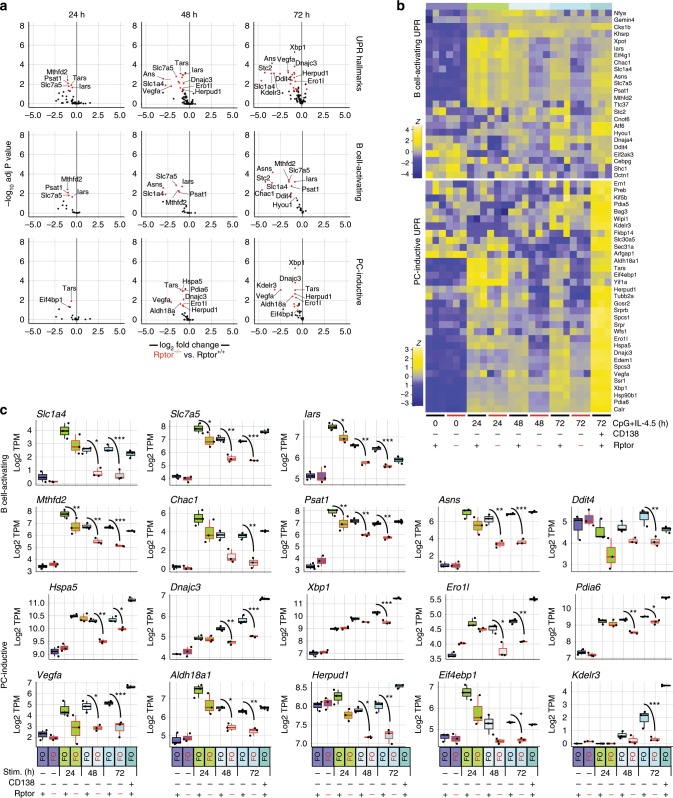
mTORC1-driven UPR gene expression in activation and differentiation. CTV-labeled follicular B cells were prepared from either CD20-TAM-Cre (Rptor^+/+^) (*n* = 3) or Rptor1^fl/fl^;CD20-TAM-Cre (Rptor^−/−^) (*n* = 3) animals after 10 days of tamoxifen diet. Sorting was performed as in Supplementary Fig. 1e. These cells were stimulated in vitro with CpG + IL-4,5 for 24, 48, or 72 h and live cells were sorted by CD138 staining. RNA-seq libraries were prepared from cDNA extracted from the resulting populations. **a** Volcano plots display differential expression of UPR hallmark genes and the gene sets defined in Fig. 3c for indicated treatment time points comparing Rptor^−/−^ populations to Rptor^+/+^ controls (group *n* = 3 animals). Genes are color coded by the differential expression statistic *p* < 0.05, log_2_ fold change > 1 (BH-adjusted *p*-value—eBayes method—Limma). **b** Expression of B-cell activating and plasma cell-inductive UPR genes as defined in Fig. 3c are shown for each animal and in vitro-treatment time point as the *z* score across each row. **c**, Log_2_ TPM magnitude of expression of selected genes in each gene set is shown as mean (line), 75%CI (box) and 95%CI (whisker), as well as individual data points (jitter) (*p < .05, **p < .01, ***p < .001; differential expression statistic: BH-adjusted *p*-value—eBayes method—Limma). Source data are provided as a Source Data file.

Among CD138^−^ cells we observed a significant and progressive loss in UPR target gene expression across all three timepoints. These genes included transcripts defined above in either activating or plasma cell-inductive conditions, comprising a wide array of genes belonging to the UPR hallmark gene set (Fig. 7a, b). Interestingly, this kinetic analysis showed that the B cell-activating UPR genes were induced at highest levels in the first 24 h of stimulation, further indicating that these loci are part of an initial wave of UPR-affiliated genes that are up-regulated coincident with early B cell activation (Fig. 7b, c). Many genes such as the amino acid transporters *Slc1a4* and *Slc7a5*, the tRNA synthase *Iars*, and the metabolic enzymes *Mthfd2*, *Chac1*, and *Psat1* all peaked in expression at 24 h and then came to be expressed intermediately at later time points in Raptor-sufficient cells. Importantly however, in Raptor-deficient cells each of these genes exhibited reduced transcript abundance at every time point. Moreover, transcripts shown above to be unique to the plasma cell-inductive program were generally seen to increase in abundance throughout the time course and were TORC1-dependent at each timepoint (Fig. 7c), further indicating that initiation of a full UPR is highly dependent on mTORC1. Theses included all the functional categories described above including chaperones (*Hspa5, Dnajc3*), disulfide bond formation (*Ero1l, Pdia6*), and protein localization and trafficking (*Kdelr3*). This kinetic analysis elucidated two distinct networks of UPR-affiliated gene expression programs that are deployed before full plasma cell differentiation. The first, in common with B cell activation, initiates a rapid homeostatic burst of enzymes needed to bring in nucleotides and amino acids and to regulate cellular processes to maintain cellular integrity. The second, unique to plasma cell inductive stimuli, results in increased mRNA abundance for loci coding for chaperones, protein localization modulators, secretion machinery, and organelle genesis. Importantly, induction of both networks was perturbed by Raptor mutation but not by Xbp1 mutation.

## Discussion

**Fig. 8:**
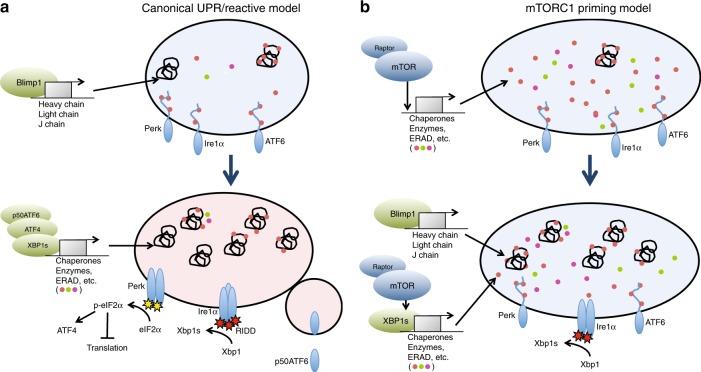
Model for mTORC1 priming of the ER preceding plasma cell differentiation. **a** The conventional model for ER remodeling during plasma cell differentiation holds that Blimp1-mediated increases in immunoglobulin expression causes the chaperone BiP to release from the luminal tails of IRE1α, allowing it to dimerize and mediate Xbp1 mRNA splicing. In this model Xbp1s is the main effector of ER remodeling. Paradoxically, with this scenario activation of PERK and ATF6 would occur by the same mechanism, causing translation inhibition and increased chance of apoptosis downstream of PERK activation. **b** Revised model wherein, preceding Blimp1 induction, mTORC1 signaling facilitates the upregulation of plasma cell-affiliated UPR genes, including chaperones, ERAD machinery, and enzymes necessary for enhancing protein secretion. The resulting “primed” ER is then better equipped to handle increased protein load following Blimp1 induction. At this point the UPR is tuned so that we see activation of the Xbp1-splicing activity of IRE1α without RIDD activity and activation of PERK.

We used global mRNA expression analyses to gain an understanding of the molecular events underlying early plasma cell genesis, before up-regulation of the Blimp1 and Xbp1s transcription factors. This work was inspired in part by past work illustrating that Blimp1-null B cells generate very small numbers of plasma cells^20^, together with our observation that BiP is induced well before Blimp1 expression^27^. By comparing mRNA signatures for in vitro-activated follicular B cells with freshly isolated MZ B cells, we learned that B cells up-regulate the transcription of multiple components of the UPR before the onset of robust antibody synthesis. Furthermore, our data support the notion that activated B cells employ mTORC1 signaling in this process, before full induction of the Blimp1/Xbp1s axis and independently of Xbp1. Thus, we propose that, while Xbp1-driven aspects of the UPR can be viewed as a reaction to increased Ig synthesis, activated B cells also anticipate increased protein synthesis by initiating the expression of many components of the UPR. We further suggest that early up-regulation of UPR components allows newborn plasma cells to maximize adaptive effects of the UPR while also minimizing pathways that lead to stress-induced attenuation of protein translation and cell death (Fig. 8). For instance, the transcription of large amounts of Dnajc3 preceding Blimp1 induction serves to attenuate the PERK-signaling axis once UPR is initiated. Additionally, priming for increased ER function through early expression of key chaperones, such as BiP (Hspa5) and GRP94 (Hsp90b1) would likely reduce ER-stress due amplification of Ig synthesis in early plasma cells.

Our results further suggest that the UPR is established in functionally disparate waves. Because activated B cells undergoing early plasma cell differentiation remain poorly defined^19,43^, and because past work on UPR regulation in plasma cells have focused mainly on Xbp1^13,15^, it has been difficult to understand how the UPR is deployed in newborn plasma cells. Our data suggest a model where activated B cells express an array of UPR-affiliated genes associated with amplification of transcription or translation including asparagine synthetase and the amino acid transporters Slc1a4 and Slc7a5; hence these gene products likely set the stage for early activation of the UPR. By contrast pre-plasma cells increase expression of a variety of transcripts encoding proteins that optimize ER function and homeostasis, such as protein chaperones and the ERAD regulators Derl3 and Edem1, all before implementation of Blimp1 and Xbp1 activity.

This model is also consistent with the dynamics of BiP up-regulation. All three ER stress sensors are activated by dissociation of BiP due to increased abundance of misfolded proteins. In this regard BiP plays dual and somewhat opposing roles in the UPR; negative regulation of ER sensors, such as IRE1α during phases of relatively low protein load in the ER, then promotion of high throughput protein synthesis by acting as a chaperone for Ig and other proteins. Transcriptional activation of many UPR targets including BiP through an alternate, mTORC1-dependent pathway, allows for priming of the system to begin to expand the ER before the onset of massive Ig synthesis.

It is becoming clear that certain B cells produce plasma cells faster and with fewer input signals than others. Such cells likely include MZ B cells together with B1 B cells and discreet subpopulations of memory B cells in the mouse^29,44,45^, and memory B cells in people^46^. Such cells likely play key and non-redundant roles in host defense, especially in scenarios, such as in sepsis where rapid and robust antibody synthesis should be highly advantageous. How these cells become primed or poised to generate plasma cells remains quite mysterious. Our results also suggest that proactive activation of the UPR underlies the rapid nature of plasma cell differentiation by MZ B cells, an idea consistent with data showing modest expansion of the ER in resting MZ B cells^47^. Of note, we have tested the idea that microbe-derived macromolecules, such as LPS drive plasma cell priming by continuously stimulating MZ B cells in situ. However, MZ B cells from germ-free mice did not differ in any of the functional assays described here (unpublished results). An alternative possibility may relate to the Notch pathway. Both the development and the maintenance of the MZ B cell pool relies heavily on Notch2 signaling^48,49^, and past work suggests that exposure of B cells to Notch2 ligands lowers activation thresholds^50^. Therefore, it is tempting to speculate that ongoing Notch2 signaling establishes unique mTOR-regulated genetic networks in MZ B cells that drive plasma cell priming. However, distinct B-cell subpopulations may also become primed for differentiation via different mechanisms. In the latter regard, B1 B cells do not require Notch signaling^48,51^, and we are unaware of data connecting the Notch pathway to memory B cell development.

mTORC1 plays diverse roles in regulating cell growth and metabolic pathways in eukaryotic cells. Additionally, evidence is mounting that mTORC1 also regulates cell type specific processes needed for unique cellular functions. Within the immune system mTORC1 coordinates signals via the T cell receptor and the IL-2 receptor to solidify regulatory T cell function through the eventual upregulation of T cell-affiliated surface receptors that confer immune suppression^52^. Within the B cell lineage recent work suggests that mTORC1 facilitates metabolic reprograming needed for B cell progenitors to proliferate in response to IL-7^53^, while also optimizing several important aspects of germinal center B cell differentiation including Bcl6 expression^54,55^. Our studies reveal additional insights into cell-type and context-specific mTORC1 functions. In this regard, past work has suggested a relationship between mTORC1 and the UPR. For instance, mutation of Tsc1, a negative regulator of mTOR activity^56^, enhances antibody secretion and improves ER biogenesis in Xbp1-deficient plasma cells^28^. Our data extend these past results by providing insights into the timing with which mTORC1 promotes UPR activation in the normal physiologic generation of antibody-secreting cells.

The connection made here between mTORC1 and transcript abundance is consistent with past work showing that mTORC1 may regulate transcription^25^. For instance, adding rapamycin to fibroblasts reduces transcript abundance for several genes linked to metabolism^57^, and mTORC1 has been proposed to promote the transcription of genetic targets of the transcription factor HIF1α, a key regulator of the cellular response to hypoxia^58^. For many such genes the mechanisms whereby mTORC1 influences mRNA abundance are likely to be indirect. In this regard, HIF1α-binding motifs are present in the promoters of numerous rapamycin-sensitive genes in fibroblasts^57^, and in yeast rapamycin affects the nuclear localization of several transcription factors (reviewed by Crespo et al.^59^). Therefore, motif analyses of the promoters of UPR-related genes in activated B cells might yield insights into the mechanisms underlying mTORC1 regulation of gene expression in early plasma cell differentiation.

In sum, our results illustrate a complex and dynamic pattern of gene and protein expression for activated B cells in the throes of early plasma cell genesis. Whereas the vast majority of past studies on early plasma cell differentiation has focused on activation of the Blimp1/IRF4/Xbp1-centric GRN, our work focuses on an earlier and largely overlooked aspect of plasma cell genesis, providing insights into a novel regulatory pathway for UPR activation and a novel function for the mTORC1 kinase. These results and the supporting data provide a resource for future identification of molecular circuits important for early and mature plasma cell function and survival that could become targets for manipulating antibody synthesis in a variety of settings.

## Methods

### Mice

C57BL/6 (C57BL/6J) were procured from Jackson Laboratories and housed in our colony at U. Penn. B6.Blimp1^+/GFP^ (Prdm1^+/GFP^)^60^ and hCD20.Tam-Cre mice^61^ were bred and housed in our animal colony. hCD20.Tam-Cre mice were generously provided by Dr. Mark Shlomchik and Xbp1^f/f^ mice were generously provided by Dr. Laurie Glimcher and were bred and housed in our animal colony^62^. All animal procedures were approved by the University of Pennsylvania Office of Regulatory Affairs.

### Cell preparation

Spleen cells were obtained by mechanical disruption of the spleen between two frosted glass slides in FACS buffer (PBS, 0.1% BSA, 1 mM EDTA) followed by filtration through 0.66 μm nitex mesh and red blood cell lysis with ACK lysis buffer. BM cells were obtained by flushing marrow from the femur and tibia of each hind leg using a syringe with 23-gauge needle and FACS buffer followed by filtration through 0.66 μm nitex mesh and red blood cell lysis with ACK lysis buffer.

### Cell culture and stimulation

Splenocytes from 10-week-old mice were sorted into complete growth media (RPMI, 10% FBS, HEPES, Penn/Strep, NEAA, Na-Pyruvate, 2-ME). Cells were plated at 1 × 10^6^ cells/ml in 96-well culture plates at 200 μl/well. Cultures were supplemented as indicated with CpG DNA (0.1 μM), IL-4 (100 ng/ml), IL-5 (100 ng/ml), and rapamycin (2 nM). Cultures were incubated at 37 °C, 5% CO_2_ for the indicated times.

### Flow cytometry and sorting

Following RBC lysis, cells were stained with Zombie-Aqua Fixable Viability kit (BioLegend) according to the manufacturer’s protocol and then stained with a cocktail of appropriate antibodies at optimized concentrations for 30 min on ice. Flow cytometry was performed using BD LSRII and LSR Fortessa analyzers and cell sorting with BD FACS Aria cell sorters. Data analysis was performed using Flowjo 8.8.7 software. Steady-state cell populations were twice-sorted by the following gating strategies as described^63,64^: Follicular B: size, singlet, live, CD19^+^, AA4.1^−^, CD23^+^/CD21/35^mid^; MZ B: size, singlet, live, CD19^+^ AA4.1^−^, CD23^−^, CD21/35^high^; Immature BM plasma cells: size, singlet, live, IgD^−^, Dump^−^, Blimp1/GFP^+^, CD138^high^, B220^+^; Long-lived BM plasma cell: size, singlet, live, IgD^−^, Dump^−^, Blimp1/GFP^+^, CD138^high^, B220^−^; LPS-induced splenic plasma cells: size, singlet, live, IgD^−^, Dump^−^, Sca1^+^, CD138^high^. Cells from in vitro stimulation cultures were sorted as shown in Supplementary Fig. 2.

### Intracellular staining

Intracellular pS6 and pAKT staining were performed using BD Cytofix and BD Phosphlow Perm Buffer III two-step protocol. Intracellular Xbp1s and BiP analyses were performed using the ThermoFisher Foxp3 Transcription Factor Staining Buffer Kit using the manufacturer’s one step intranuclear, 96-well plate protocol.

### Western blotting

Sorted viable cells were lysed in RIPA lysis buffer and protein was quantified by BCA assay. 20 μg protein was loaded and run on SDS–PAGE 4–20% gradient gel. Proteins were transferred to nitrocellulose at 200 mA for 3 h. Primary antibodies were incubated at 4 °C overnight 1:1000 in 5% BSA in Tris-buffered saline + 1% Tween 20 (TBST). Secondary antibodies were incubated 1 h at room temperature 1:3000 in 5% milk TBST.

### Antibodies

CD23 (B3B4)-PE and -BV421[1:200], TCRβ (H57-597)-PE[1:400], Ter-119-PE[1:400], CD138 (281-2)-PE[1:400] and XBP1s (Q3-695)-PE-CF594[1:200] were purchased from BD Biosciences. IgD (11-26.2a)-APC-Cy7[1:200], CD4 (GK1.5)-PE-Cy7[1:400], Ter-119-PE-Cy7[1:400], F4-80 (BM8)-PE-Cy7[1:400], Sca-1 (D7)-BV605[1:200], B220 (RA3-6B2)-BV421[1:200], and CD138 (281-2)-BV605[1:400] were purchased from BioLegend. ATF-4 (D4B8)[1:1000], XBP1s (E8Y5F) [1:1000], p58IPK (C56E7)[1:1000], ATF-6 (D4Z8V) [1:1000], BiP (C50B12)-PE[1:200], pAKT (S473) (D9E)-PE[1:100] and pS6(S235/236) (D57.2.2E)-PE-Cy7[1:200] were purchased from Cell Signaling Technology. AA4.1-APC[1:100], CD21/35 (8D9)-PE-Cy7[1:200], IgM (11/41)-PerCP-ef710[1:200], and CD8α (53-6.7)-PE and -PE-Cy7[1:400] were purchased from eBioscience (ThermoFisher). CD19 (6D5)-APC-Cy5.5[1:150] and F4-80 (BM8)-PE[1:400] were purchased from Invitrogen. B220 (RA3-6B2)-APC[1:200] was purchased from Tonbo biosciences.

### RNA-sequencing

Cell populations were sorted directly into Trizol with 0.5% 2-ME and held at −80 °C until RNA preparation. RNA was prepared by published Trizol RNA-extraction protocol (Thermo-Fisher Scientific). RNA was co-precipitated using glycogen as a carrier. RNA was quantified using Qubit RNA high sensitivity fluorometric assay. cDNA was prepared using Takara Clonetech SMART-Seq® v4 Ultra® Low Input RNA Kit according to protocol using 500–1000 ng RNA as input. cDNA was quantified and qualified using HSDNA assay on an Agilent 2200 bioanalyzer. RNA-seq libraries were constructed using the Illumina Nextera XT kit with 150 ng cDNA input. Libraries were quality controlled and quantified by bioanalyzer and pooled at equal molar ratio preceding sequencing Illumina Hiseq (50 bp SE v4 high output) and Illumina Nextseq500 (75 bp SE v2) machines.

### Pseudoalignment and gene expression

Transcript abundance was computed by pseudoalignment with Kallisto^65^. Transcript per million (TPM) values were then normalized and fitted to a linear model by empirical Bayes method with the Voom and Limma R packages^66,67^ and differential gene expression was defined as a Benjemini and Hochberg corrected *p*-value of < 0.05 and fold change > 2 unless otherwise noted.

### GO cluster enrichment

GO analysis was performed using the DAVID Bioinformatics Resources 6.8, NIAID, NIH. using an enrichment score cutoff of 1.3 for clusters of GO terms, choosing the term with highest number of represented genes as founder term for each cluster. Non-descriptive or overly general terms were disregarded in favor of the term with the next highest number of genes represented^68,69^.

### Gene-set enrichment analysis

GSEA was performed using the Broad institute GSEA java tool with both curated genesets and our described investigator-defined genesets^36,70^.

### ELISpot

ELISpot assay plates (Millipore MSIPN4W50) were coated with capture goat anti-Ig-heavy/Ig-light antibody (Southern Biotech 1010-01) at 10 μg/ml and blocked in complete growth media (RPMI, 10% FBS, HEPES, Penn/Strep, NEAA, Na-Pyruvate, 2-ME). Cells were plated in complete growth media at indicated cell number or at 10^5^ cells/well and serially diluted and incubated overnight. Biotinylated goat anti-IgH/IgL (Southern Biotech 1010-08) capture antibody was used at 0.1 μg/ml. Extraavidin alkaline phosphatase was used at 1:10,000 final dilution. Spots were developed using BCIP/NBT liquid substrate and 1 M sodium phosphate stop solution. Image capture, counting, and quality control was performed using the CTL Immunospot Analyzer and software (Cellular Technologies Limited).

### Reporting summary

Further information on research design is available in the Nature Research Reporting Summary linked to this article.

## Supplementary information

Supplementary Information

Reporting Summary

## Data Availability

All RNAseq data have been deposited in the GEO database under the accession code GSE141423. All other data for this study are available from the corresponding author upon request.

## Source data

Source Data
