# Supplementary material for: mTORC1 coordinates an immediate unfolded protein response-related transcriptome in activated B cells preceding antibody secretion
Source: Nat Commun. 2020 Feb 5;11:723. doi: 10.1038/s41467-019-14032-1 (PMC7002553; doi:10.1038/s41467-019-14032-1)
Supplement: Supplementary file 1 — Supplementary Information [file 41467_2019_14032_MOESM1_ESM.pdf]

mTORC1 coordinates an immediate unfolded protein response-related transcriptome in activated B cells prior to antibody secretion

Brian T. Gaudette,[1] Derek D. Jones,[1] Alexandra Bortnick,[1] Yair Argon,[1,2] and David Allman[1*]

Affiliations:

[1]The Department of Pathology and Laboratory Medicine, Perelman School of Medicine at the University of Pennsylvania, Philadelphia, PA, USA, 19104

[2]The Children's Hospital of Philadelphia, Philadelphia, PA, USA, 19104

[*] Corresponding author: David Allman, University of Pennsylvania, 36th & Hamilton Walk, 230 John Morgan Building, Philadelphia, PA 19104-6082, E-mail: dallman@pennmedicine.upenn.edu

Supplementary Figures

Fig 1: Control of B cell activation versus differentiation.

Fig 2: Resting marginal zone B cells possess a unique transcriptome associated with enhanced protein synthesis and cellular activation.

Fig 3: Sort gates for purifying Blimp1$^{+/-}$ activated follicular B cells.

Fig 4: Expression of UPR gene targets in in-vivo activated B cells.

Fig 5: Robust activation of the UPR in Xbp1-deficient cells.

Fig 6: Efficient deletion of Rptor impairs differentiation but does not impact viability.

Table 1: Functional heterogeneity in UPR program genes.

Supplementary Figures:

Supplementary Figure 1

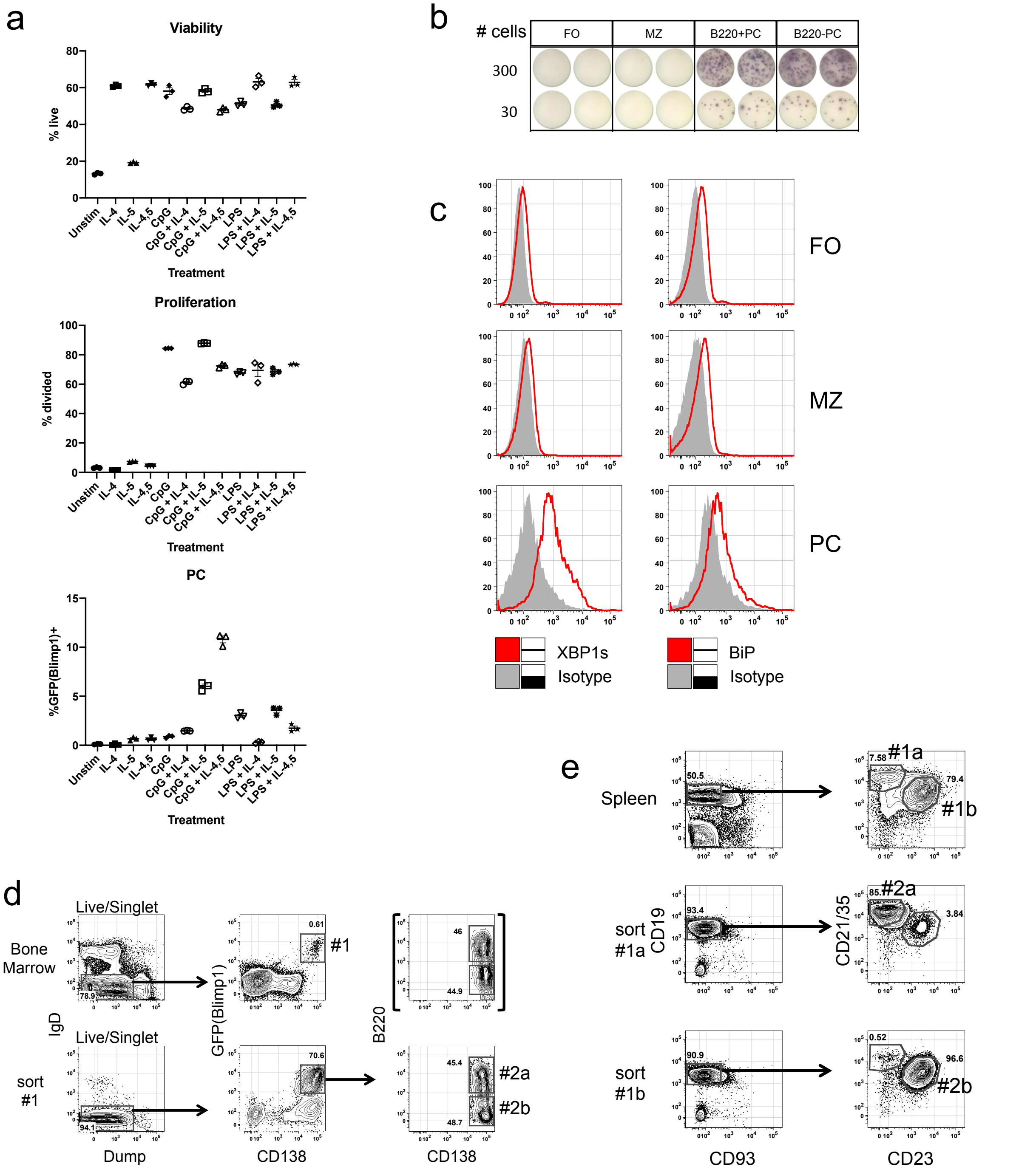

Supplementary Figure 1: Control of B cell activation versus differentiation. **a**, Splenic follicular B cells were CTV labeled, purified by CD23 column selection from B6.Blimp1$^{+/GFP}$ mice, stimulated in culture as indicated and analyzed by flow cytometry. Summary data is shown as individual data points, mean and SEM. **b**, Splenic follicular (FO), marginal zone (MZ), and bone

marrow immature (B220+) and long-lived (B220-) plasma cells (PC) were sorted from adult female C57BL/6 mice. The indicated number of cells from each population were added to ELISPOT plates coated with anti-mouse heavy and light chain polyclonal antibody. **c**, C57BL6 mouse spleens were stained for intracellular BiP and XBP1s as well as relevant isotype controls and analyzed by flow cytometry. Representative plots from 3 independent replicates are shown. **d.** Gating and sorting strategy is shown for bone marrow plasma cell subsets indicating where primary and secondary sort populations were gated for B220+ PC (#2a), and B220- PC (2b). **e.** Gating and sorting strategy is shown for splenic B cell populations indicating where primary and secondary sort populations were gated for MZ B cell (#2a) and follicular B cell (#2b) groups. Source data are provided as a Source Data file.

Supplementary Figure 2

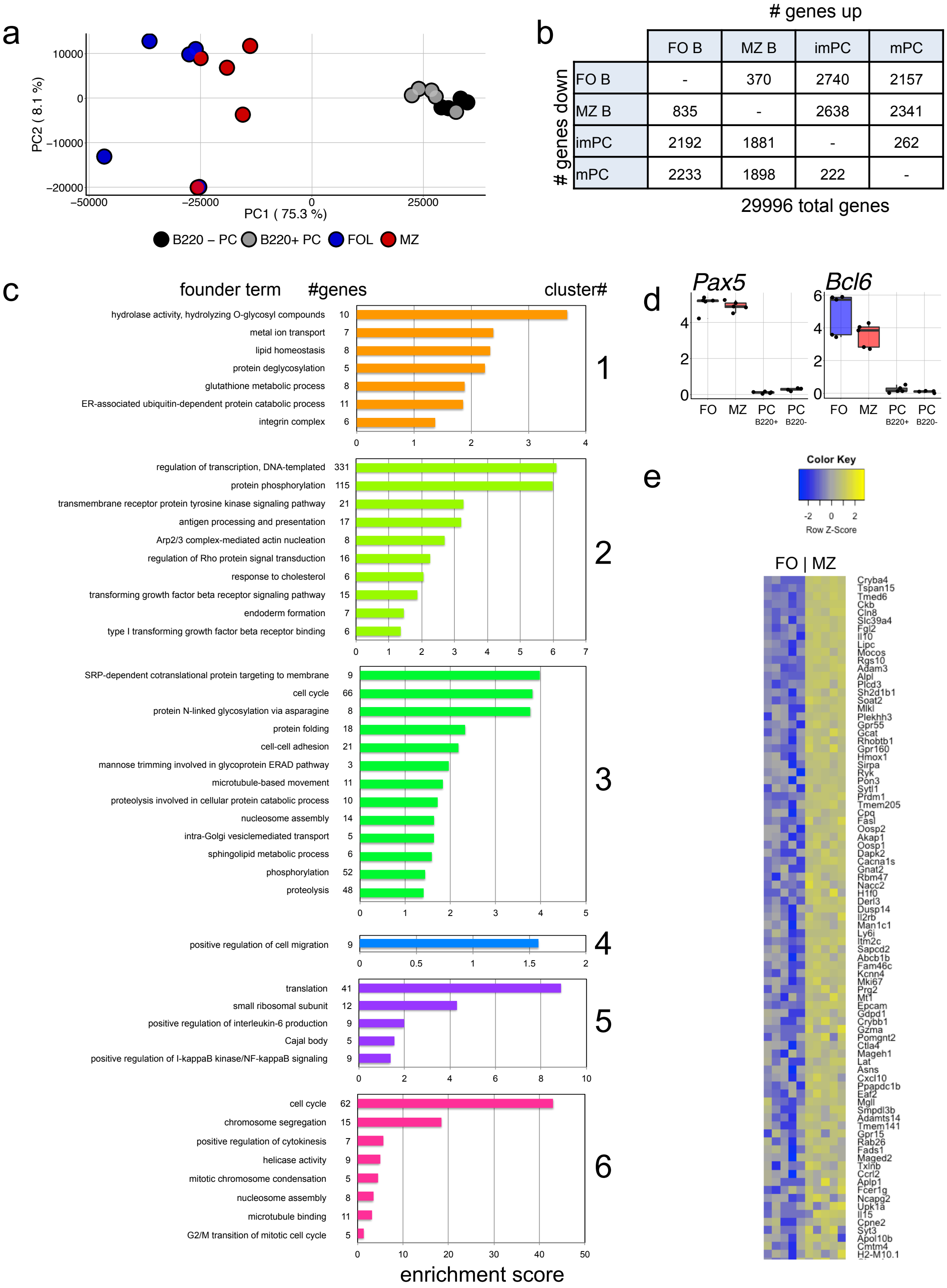

Supplementary Figure 2: Resting MZ B cells possess a unique transcriptome associated with enhanced protein synthesis and cellular activation. **a,** PCA analysis of RNA-seq data derived from the indicated cell populations from 4-5 individual mice. **b,** Numbers of differentially expressed

genes in each population established using a 2-fold cut-off and adjusted p value <0.05 (BH-adjusted P-value - eBayes method). **c**, GO term clustering analysis for all co-regulated gene clusters from Fig 2A. Indicated is the founder term for each GO term cluster followed by the number of genes represented in that term. Bar length is an indication of the enrichment score for the GO cluster. **d,** The leading-edge gene expression is shown by Z score for the PC-specific gene signature showing the most differentially expressed genes in the signature between MZ and follicular B cells.

Supplementary Figure 3

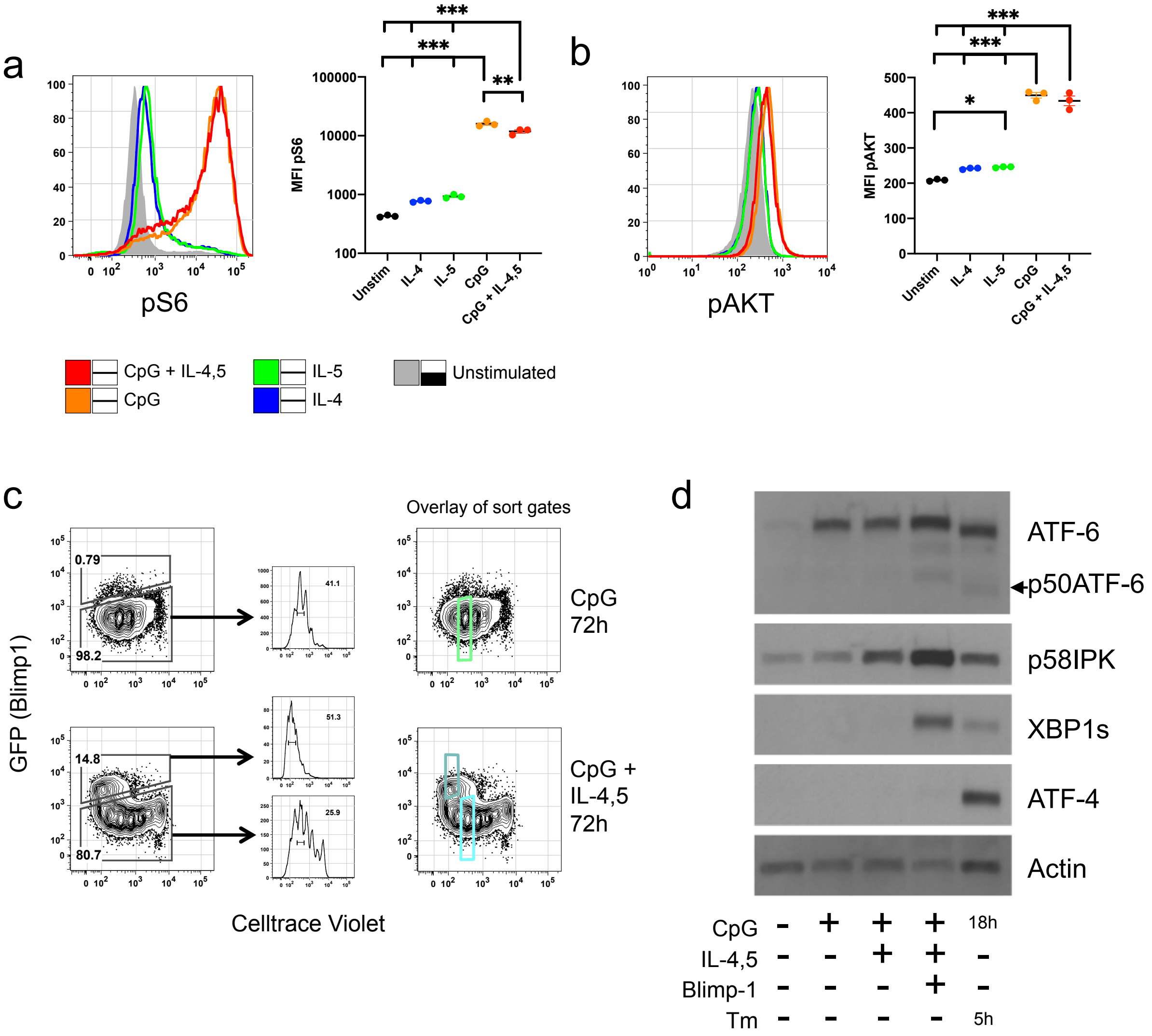

Supplementary Figure 3: **a**,**b**, CD23 selected follicular B cells were stimulated as indicated for 18 hours prior to analysis by intracellular flow cytometry. Summary data is shown as individual data points, mean and SEM. (*$p<0.05$, **$p<0.01$, ***$p<0.001$ One-way ANOVA, Tukey post-test). **c**, Sort gates for purifying Blimp1$^{+/-}$ activated follicular B cells. CTV-labeled follicular B cells from adult female B6.Blimp1$^{+/GFP}$ mice were stimulated for 72 hours with CpG +/- IL-4 and IL-5. All plots show viable cells only. **d**, CD23 selected follicular B cells from B6.Blimp1$^{+/GFP}$ mice were stimulated for 72 hours as indicated prior to sorting on divided Blimp^{+} and Blimp^{-} fractions and analysis by western blot. Tunicamycin (Tm) treated follicular B cells cells were activated with CpG overnight and then treated with 1 µg/mL tunicamycin for 5 hours prior to lysis. Source data are provided as a Source Data file.

Supplementary Figure 4

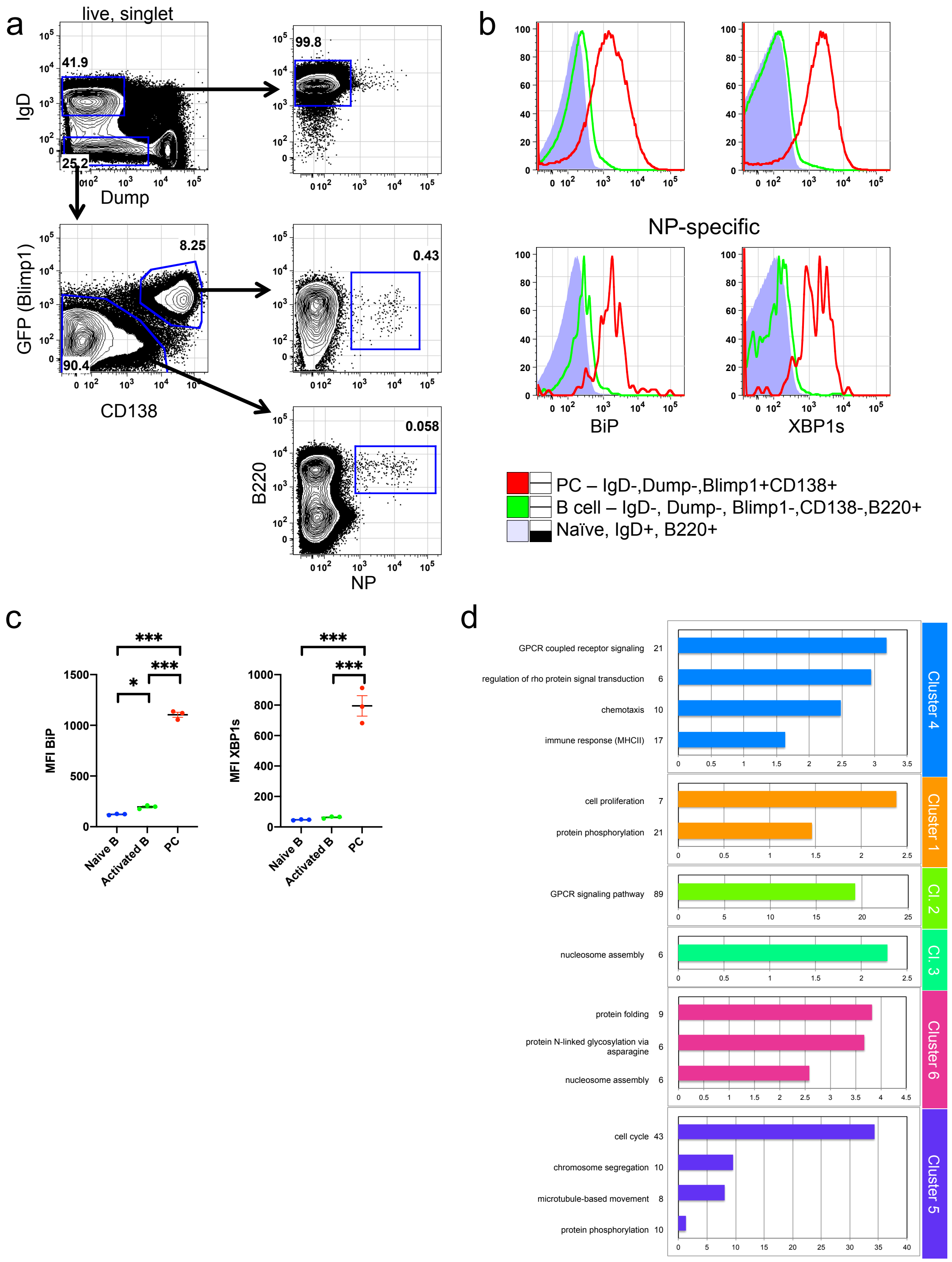

Supplementary Fig 4: Expression of UPR gene targets in in-vivo activated B cells. B6.Blimp1$^{+/GFP}$ mice were immunized with 50 µg NP-LPS 4 days prior to splenocyte harvest. Each population was sorted as gated and then sorted again to ensure purity of rare populations. **a**, Gating

strategy is show for isolation by FACS sorting of NP-Specific PCs and B cells as well as naïve B cells. **b**, Intracellular staining of BiP and XBP1s is shown for each group 4 days post immunization with NP-LPS. Top row displays all PCs and B cells in the parent gate while the bottom indicates the staining for antigen specific cells. **c**, Quantification of three animal replicates of experiment in b is shown. Summary data is shown as individual data points, mean and SEM. (*$p<0.05$, **$p<0.01$, ***$p<0.001$ One-way ANOVA, Tukey post-test) **d**, GO term clustering analysis for all co-regulated gene clusters from Fig 5a. Indicated is the founder term for each GO term cluster followed by the number of genes represented in that term. Bar length is an indication of the enrichment score for the GO cluster. Source data are provided as a Source Data file.

Supplementary Figure 5

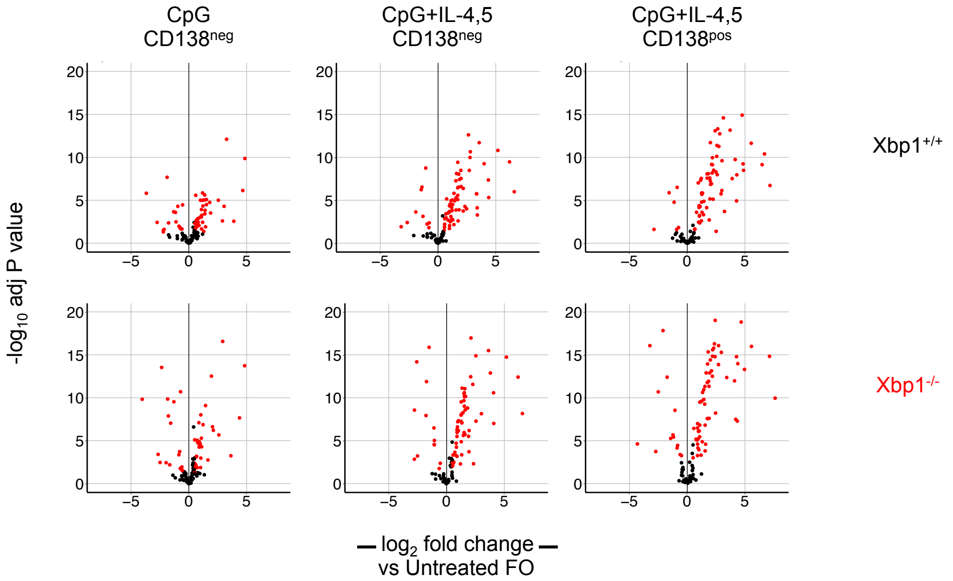

Supplementary Figure 5: Robust activation of the UPR in Xbp1-deficient cells. RNA-seq data from CpG or CpG + IL-4,5 treated, Xbp1-sufficient (Xbp1$^{+/+}$) and Xbp1–deficient (Xbp1$^{-/-}$) follicular B cells for UPR hallmark genes is displayed as volcano plots. Adjusted P value is the –log10 transformed differential expression statistic, BH-adjusted P-value - eBayes method – Limma. Genes coded in red indicate adjusted p value <0.05 and log2 fold change > 1.

Supplementary Figure 6

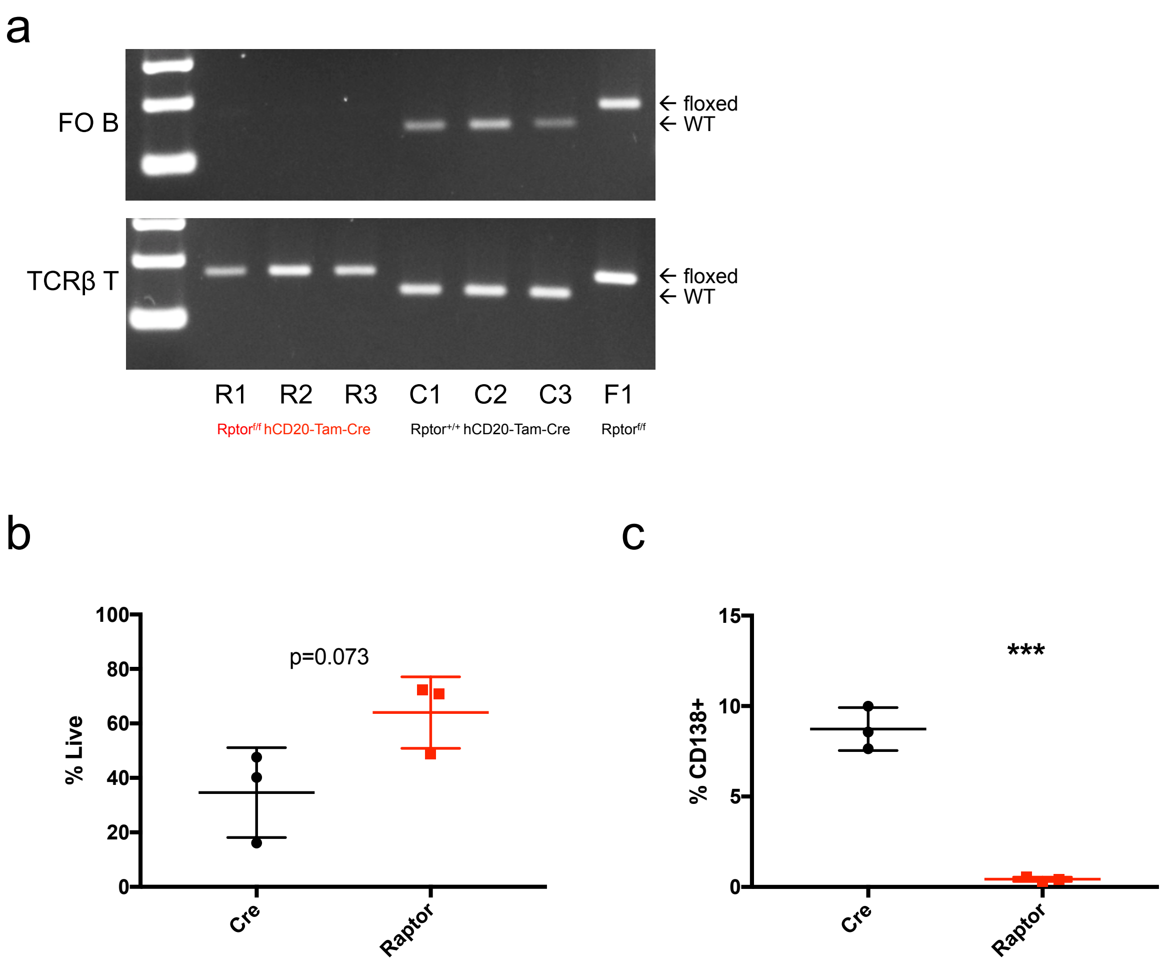

Supplementary Figure 6: Efficient deletion of Rptor impairs differentiation but does not impact viability. **a**, CD20-TAM-Cre;Rptor$^{fl/fl}$ (R1-3) and CD20-TAM-Cre;Rptor$^{+/+}$ (C1-3) were treated with tamoxifen for 10 days and genomic DNA from purified follicular B cells (CD19^{+}, AA4.1^{-}, CD23^{+}, CD21/35^{int}) and CD19^{-}, TCRβ^{+} T cells was used to detect WT and floxed Rptor by PCR. Note loss of PCR product in CD19^{+} CD20-TAM-Cre; Rptor$^{fl/fl}$ cells. Lane 7 (F1) is PCR product from a Rptor$^{fl/fl}$ animal lacking a Cre allele. **b-c**, Raptor-sufficient and deficient follicular B cells were stimulated with CpG + IL-4,5 for 72 hours were analyzed for viability and CD138 expression by flow cytometry. Summary data is shown as individual data points, mean and SEM (*p<0.05, **p<0.01, ***p<0.001 Students T test). Source data are provided as a Source Data file.

Supplementary Tables

Supplementary Table 1

**B cell activating UPR**		**PC-inductive UPR**	
chaperone	Dnaja4	chaperone	Bag3
hypoxia	Hyou1		Dnajc3
ion transport	Chac1		Fkbp14
	Stc2		Hspa5
microtubule regulation	Dctn1		Hsp90b1
transcriptional regulation	Cebpg		Herpud1
	Cnot6	hypoxia	Vegfa
	Gemin4	ion transport	Slc30a5
	Nfya	microtubule regulation	Kif5b
translational regulation	Asns		Tubb2a
	Eif4g1	transcriptional regulation	Aldh18a1
	Iars		Preb
	Khsrp	translation regulation	Eif4ebp1
	Psat1		Tars
	Slc1a4	UPR regulation	Xbp1
	Slc7a5		Ern1
	Ttc37	autophagy	Wipi1
	Xpot	calcium-dependent protein binding	Wfs1
UPR regulation	Atf6		Calr
	Eif2ak3	endocytosis	Arfgap1
cell cycle	Cks1b	ER-golgi localization	Kdelr3
mTORC1 regulation	Ddit4		Sec31a
oxidoreductase	Mthfd2		Yif1a
signaling	Shc1		Gosr2
		ERAD	Edem1
		isomerase	Ero1l
			Pdia5
			Pdia6
		protein trafficking to ER	Spcs1
			Spcs3
			Srpr
			Srprb
			Ssr1

Supplementary Table 1: Functional heterogeneity in UPR program genes. B cell activating and plasma cell inductive UPR gene segments defined in Figure 3 were grouped by cellular and molecular function. Using GO molecular function terms and NCBI gene descriptions genes were functionally identified and then grouped into connected categories. Color coding matches pie charts in Fig 4b.
