# Supplementary material for: mTORC1 coordinates an immediate unfolded protein response-related transcriptome in activated B cells preceding antibody secretion
Source: Nat Commun. 2020 Feb 5;11:723. doi: 10.1038/s41467-019-14032-1 (PMC7002553; doi:10.1038/s41467-019-14032-1)
Supplement: Supplementary file 2 — Reporting Summary [file 41467_2019_14032_MOESM2_ESM.pdf]

nature research

Corresponding author(s): David M. Allman

Last updated by author(s): Dec 5, 2019

Reporting Summary

Nature Research wishes to improve the reproducibility of the work that we publish. This form provides structure for consistency and transparency in reporting. For further information on Nature Research policies, see Authors & Referees and the Editorial Policy Checklist .

Statistics

For all statistical analyses, confirm that the following items are present in the figure legend, table legend, main text, or Methods section.

n/a	Confirmed	
☐	☒	The exact sample size (*n*) for each experimental group/condition, given as a discrete number and unit of measurement
☐	☒	A statement on whether measurements were taken from distinct samples or whether the same sample was measured repeatedly
☐	☒	The statistical test(s) used AND whether they are one- or two-sided<br>*Only common tests should be described solely by name; describe more complex techniques in the Methods section.*
☐	☒	A description of all covariates tested
☐	☒	A description of any assumptions or corrections, such as tests of normality and adjustment for multiple comparisons
☐	☒	A full description of the statistical parameters including central tendency (e.g. means) or other basic estimates (e.g. regression coefficient) AND variation (e.g. standard deviation) or associated estimates of uncertainty (e.g. confidence intervals)
☒	☐	For null hypothesis testing, the test statistic (e.g. *F*, *t*, *r*) with confidence intervals, effect sizes, degrees of freedom and *P* value noted<br>*Give P values as exact values whenever suitable.*
☐	☒	For Bayesian analysis, information on the choice of priors and Markov chain Monte Carlo settings
☒	☐	For hierarchical and complex designs, identification of the appropriate level for tests and full reporting of outcomes
☒	☐	Estimates of effect sizes (e.g. Cohen's *d*, Pearson's *r*), indicating how they were calculated

Our web collection on statistics for biologists contains articles on many of the points above.

Software and code

Policy information about availability of computer code

Data collection	RNA-seq data were generated on the Illumina Hiseq and NextSeq platforms. Fastq sequence data were generated from BCL files through the standard Illumina pipeline.<br>Flow cytometry data was collected on BD FACSAria sorters, and BD LSRII and LSRFortessa analyzers.
Data analysis	All downstream RNA-seq analyses were performed with open source software. TPM data were generated with Kallisto v 0.43.1, differential expression was assessed with limma 3.40.2 (Voom normalization and linear modeling and hypothesis testing). All RNA-seq data display and additional statistics were performed within the R statistical environment v 3.6.0.<br>Flow cytometry data were analyzed with Flowjo v 8.8.7<br>Additional graphical and statisitcal analyses were performed within Graphpad Prism v 8.

For manuscripts utilizing custom algorithms or software that are central to the research but not yet described in published literature, software must be made available to editors/reviewers. We strongly encourage code deposition in a community repository (e.g. GitHub). See the Nature Research guidelines for submitting code & software for further information.

Data

Policy information about availability of data

All manuscripts must include a data availability statement. This statement should provide the following information, where applicable:

- Accession codes, unique identifiers, or web links for publicly available datasets
- A list of figures that have associated raw data
- A description of any restrictions on data availability

Data availability: All RNAseq data have been deposited in the GEO database under the accession code GSE141423 [https://www.ncbi.nlm.nih.gov/geo/query/acc.cgi?acc=GSE141423]. All other data for this study are available from the corresponding author upon request.

Field-specific reporting

Please select the one below that is the best fit for your research. If you are not sure, read the appropriate sections before making your selection.

- [x] Life sciences
- [ ] Behavioural & social sciences
- [ ] Ecological, evolutionary & environmental sciences

For a reference copy of the document with all sections, see nature.com/documents/nr-reporting-summary-flat.pdf

Life sciences study design

All studies must disclose on these points even when the disclosure is negative.

Sample size	Based on past very similar experiments in inbred mice.
Data exclusions	No completed data were excluded from the findings
Replication	We repeated experiments on average 2-3 times with biological replicates for each individual experiment.
Randomization	Mice were grouped according to genotype and no additional treatments were performed so randomization was not appropriate. Wherever possible hetXhet breeding was used to ensure that all genotypes were found amongst littermate groups.
Blinding	Not relevant.

Reporting for specific materials, systems and methods

We require information from authors about some types of materials, experimental systems and methods used in many studies. Here, indicate whether each material, system or method listed is relevant to your study. If you are not sure if a list item applies to your research, read the appropriate section before selecting a response.

Materials & experimental systems

n/a	Involved in the study	
[ ]	[x]	Antibodies
[x]	[ ]	Eukaryotic cell lines
[x]	[ ]	Palaeontology
[ ]	[x]	Animals and other organisms
[x]	[ ]	Human research participants
[x]	[ ]	Clinical data

Methods

n/a	Involved in the study	
[x]	[ ]	ChIP-seq
[ ]	[x]	Flow cytometry
[x]	[ ]	MRI-based neuroimaging

Antibodies

Antibodies used	Antibodies (clone) [Dilution] and providers:<br>CD23 (B3B4)-PE and –BV421[1:200], TCRβ (H57-597)-PE[1:400], Ter-119-PE[1:400], CD138 (281-2)-PE[1:400] and XBP1s (Q3-695)-PE-CF594[1:200] were purchased from BD Biosciences. IgD (11-26.2a)-APC-Cy7[1:200], CD4 (GK1.5)-PE-Cy7[1:400], Ter-119-PE-Cy7[1:400], F4-80 (BM8)-PE-Cy7[1:400], Sca-1 (D7)-BV605[1:200], B220 (RA3-6B2)-BV421[1:200], and CD138 (281-2)-BV605[1:400] were purchased from BioLegend. ATF-4 (D4B8)[1:1000], XBP1s (E8Y5F) [1:1000], p58IPK (C56E7) [1:1000], ATF-6 (D4Z8V) [1:1000], BiP (C50B12)-PE[1:200], pAKT (S473) (D9E)-PE[1:100] and pS6(S235/236) (D57.2.2E)-PE-Cy7[1:200] were purchased from Cell Signaling Technology. AA4.1-APC[1:100], CD21/35 (8D9)-PE-Cy7[1:200], IgM (11/41)-PerCP-ef710[1:200], and CD8α (53-6.7)-PE and –PE-Cy7[1:400] were purchased from eBioscience (ThermoFisher). CD19 (6D5)-APC-Cy5.5[1:150] and F4-80 (BM8)-PE[1:400] were purchased from Invitrogen. B220 (RA3-6B2)-APC[1:200] was purchased from Tonbo biosciences.
Validation	All reagents were purchased from established vendors.

Animals and other organisms

Policy information about studies involving animals; ARRIVE guidelines recommended for reporting animal research

Laboratory animals	Mus musculus:<br>Strain: C57BL/6 - age 10-14 weeks, equal numbers male/female where possible<br>Substrains: B6.BlimpGFP, B6.hCD20-TamCre, Xbp1-flox, Raptor-flox<br>B6.BlimpGFP mice are maintained as B6.Blimp+/GFP and continuously bred to B6 dams.<br>Xbp1-flox and Raptor-flox mice were bred to B6.hCD20-TamCre mice as flox-het.cre to flox-het breeders in order to insure appropriate littermate controls. All control data come from Cre expressing animals lacking floxed alleles to account for cre recombinase activity effects

Wild animals	n/a
Field-collected samples	n/a
Ethics oversight	All studies were approved by the regulatory affairs (IACUC) office at the University of Pennsylvania.

Note that full information on the approval of the study protocol must also be provided in the manuscript.

Flow Cytometry

Plots

Confirm that:

- [x] The axis labels state the marker and fluorochrome used (e.g. CD4-FITC).
- [x] The axis scales are clearly visible. Include numbers along axes only for bottom left plot of group (a 'group' is an analysis of identical markers).
- [x] All plots are contour plots with outliers or pseudocolor plots.
- [x] A numerical value for number of cells or percentage (with statistics) is provided.

Methodology

Sample preparation	Spleens were disrupted mechanically using frosted glass slides and filtered through 68 micron nylon mesh, RBC lysis was performed with ACK buffer and where applicable cells were labeled with Celltrace Violet proliferation dye.<br>Bone marrow was flushed from leg bones with 23 gauge needles and filtered through 68 micron nylon mesh, and RBCs were lysed with ACK buffer.
Instrument	Sorting: BD FACSAria<br>Analysis: BD LSR II, BD LSR Fortessa
Software	Flowjo v 8.8.7
Cell population abundance	Plasma cell: initial: ~0.1% of total bone marrow. Sorted twice to ensure purity. Samples were initially sorted on total plasma cells then on subset plasma cells on second sort. Initial sort yielded ~70% purity, second sort yielded >95% purity.<br>Marginal zone B cells: Initial ~3% total spleen. Initial sort yielded ~80% purity, second sort yielded >95% purity.<br>Follicular B cells: Initial ~30% total spleen. Initial sort yielded ~95% purity, second sort yielded >95% purity.<br>All final sorts were checked for purity by sorting an additional aliquot for re-run.<br>Sorting strategy and representative sort data from a study mouse are shown in Supplementary Figure 1.
Gating strategy	Plasma cell: FSC/SSC size gate (lymphocyte to large lymphocyte), singlet, Live/Dead negative, IgD-, Dump(CD4, CD8a, Ter-119, F4/80)-, GFP(Blimp1)+, CD138high, B220+/-<br>Follicular B cell: FSC/SSC size gate (lymphocyte), singlet, Live/Dead negative, CD19+, CD93-, CD23+, CD21int<br>Marginal Zone B cell: FSC/SSC size gate (lymphocyte), singlet, Live/Dead negative, CD19+, CD93-, CD23-, CD21high

- [x] Tick this box to confirm that a figure exemplifying the gating strategy is provided in the Supplementary Information.
